# Condensin Smc2-Smc4 Dimers Are Flexible and Dynamic

**DOI:** 10.1016/j.celrep.2016.01.063

**Published:** 2016-02-18

**Authors:** Jorine M. Eeftens, Allard J. Katan, Marc Kschonsak, Markus Hassler, Liza de Wilde, Essam M. Dief, Christian H. Haering, Cees Dekker

**Affiliations:** 1 Department of Bionanoscience, Kavli Institute of Nanoscience Delft, Delft University of Technology, Delft 2628 CJ, the Netherlands; 2 Cell Biology and Biophysics Unit, European Molecular Biology Laboratory (EMBL), 69117 Heidelberg, Germany

## Abstract

Structural maintenance of chromosomes (SMC) protein complexes, including cohesin and condensin, play key roles in the regulation of higher-order chromosome organization. Even though SMC proteins are thought to mechanistically determine the function of the complexes, their native conformations and dynamics have remained unclear. Here, we probe the topology of Smc2-Smc4 dimers of the *S. cerevisiae* condensin complex with high-speed atomic force microscopy (AFM) in liquid. We show that the Smc2-Smc4 coiled coils are highly flexible polymers with a persistence length of only ∼4 nm. Moreover, we demonstrate that the SMC dimers can adopt various architectures that interconvert dynamically over time, and we find that the SMC head domains engage not only with each other, but also with the hinge domain situated at the other end of the ∼45-nm-long coiled coil. Our findings reveal structural properties that provide insights into the molecular mechanics of condensin complexes.

## Highlights

• The conformation and dynamics of SMC dimers were imaged with high-speed AFM
• The heads of Smc2 and Smc4 engage with each other and with the hinge dynamically
• The coiled coils of Smc2 and Smc4 are flexible and show extensive fluctuations in time

## Graphical Abstract

**Figure:**
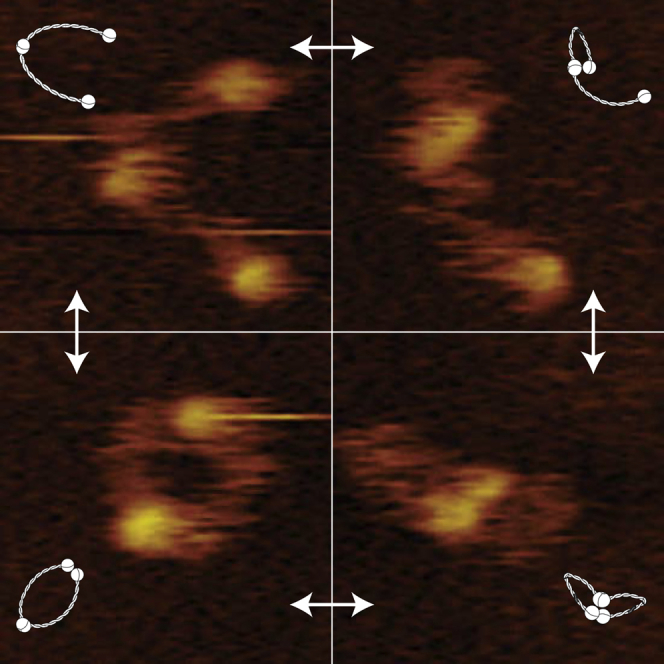

## Teaser

Eeftens et al. probe the topology and dynamics of condensin’s Smc2-Smc4 dimers with high-speed AFM in liquid. They find that the Smc2-Smc4 coiled coils are remarkably flexible and do adopt various conformations that interconvert dynamically over time.

## Introduction

**Figure 1:**
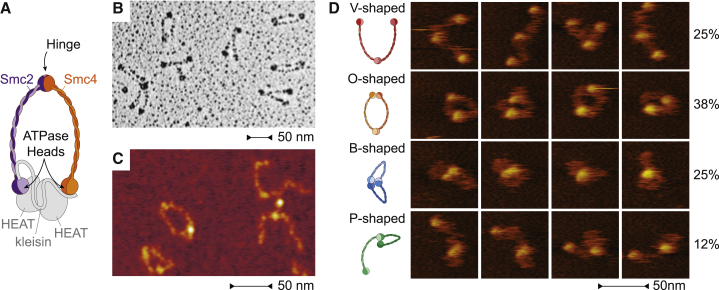
*S. cerevisiae* Smc2-Smc4 Dimers Adopt a Variety of Conformations (A) Cartoon of the eukaryotic condensin complex. Smc2 and Smc4 heterodimerize via their hinge domains. The kleisin subunit associates with the Smc2 and Smc4 ATPase head domains to create a ring-like structure and recruits two additional subunits (shown in gray, not studied here). (B) Example image of Smc2-Smc4 dimers imaged by rotary shadowing EM. (C) Example image of Smc2-Smc4 dimers imaged by dry AFM. (D) Example images of different conformational classes of Smc2-Smc4 dimers from high-speed liquid AFM movies. The frequency of each conformational class (as fraction of 1,795 total frames from 18 movies) is indicated. V-shaped, SMCs are connected at the hinge but the heads are not engaged; O-shaped, the heads are engaged with each other; B-shaped (butterfly), both heads are engaged with the hinge; P-shaped, one of the heads is engaged with the hinge.

Cohesin and condensin protein complexes play central roles in many aspects of chromosome biology, including the segregation of sister chromatids during cell divisions, compaction of chromosomes, and regulation of gene expression during interphase (reviewed in Aragon et al., 2013, Hirano, 2006). Although functionally different, cohesin and condensin have similar architectures: both complexes are composed of two different SMC subunits and a subunit of the kleisin protein family. Together, these three proteins form a ring-like structure that is conserved from bacteria to eukaryotes. The protein chain of each SMC protein folds back onto itself to form an ∼45-nm-long antiparallel coiled coil, which connects a globular “hinge” domain at one end to an ATPase “head” domain, created by the association of N- and C-terminal protein sequences, at the other end (Figure 1A). Two SMC proteins form a heterodimer by the association of their hinge domains: Smc1-Smc3 in the case of cohesin and Smc2-Smc4 in the case of condensin (Anderson et al., 2002). In addition, the head domains of the two SMC subunits can associate in the presence of ATP. The functional roles of ATP binding-mediated dimerization and hydrolysis-dependent dissociation of the two head domains have remained largely unclear. Both cohesin and condensin have been suggested to bind to chromosomes by encircling chromatin fibers topologically within their SMC-kleisin rings (Cuylen et al., 2011, Haering et al., 2008).

The conformation and dynamics of SMC dimers are of great importance, since they are thought to mechanistically determine the biological function of all SMC protein complexes. Accordingly, there have been numerous efforts to gain insight into the configuration of the SMC dimers. Electron microscopy (EM) images of cohesin complexes suggest that the Smc1–Smc3 coiled coils emerge from the hinge domain in an open conformation, resulting in V- or O-shaped arrangements with the two coils separated along most of their lengths (Anderson et al., 2002, Haering et al., 2002, Huis in ’t Veld et al., 2014). V-shaped conformations were also observed for condensin’s Smc2-Smc4. However, in a large fraction of molecules the Smc2-Smc4 coiled coils seemed to align, resulting in rod- or I-shaped rather than V-shaped conformations (Anderson et al., 2002, Yoshimura et al., 2002). Support to the notion that condensin’s SMC coiled coils tightly associate with each other came from a recent crystal structure of the Smc2-Smc4 hinge domains and parts of the adjacent coiled coils, as well as from chemical cross-linking experiments (Barysz et al., 2015, Soh et al., 2015). Small Angle X-ray Scattering (SAXS) experiments implied that also the SMC subunits of cohesin and prokaryotic SMC complexes form I-shaped molecules in solution (Soh et al., 2015). These contradicting results indicate that it is still unclear which configurations SMC dimers adopt in vivo, and under which circumstances conformational changes might occur. The major disadvantages of all methods that have so far been used to study the configuration of SMC molecules are that they probed the protein structure either in highly artificial environments (e.g., dried in vacuum or packed into a crystal lattice) or in a kinetically trapped state (e.g., by cross-linking).

Atomic force microscopy (AFM) has proved to be a powerful tool to visualize biomolecules and to study their mechanical properties at nanometer resolution without the need for labeling. Importantly, it can also be carried out in aqueous solution under physiological conditions. Recent technical advances have made it possible to observe single molecules in action with high-speed AFM, reaching frame rates of up to 20 frames per second and thereby allowing imaging in real time (Ando et al., 2001, Katan and Dekker, 2011). Here, we use high-speed AFM in liquid, in combination with supporting data from EM and dry AFM, to probe the structural arrangement and dynamics of condensin’s Smc2-Smc4 dimers under physiological conditions. We show that the coiled coils are remarkably flexible, allowing the molecules to adopt various conformations that change over time. We furthermore find that, even in the absence of ATP or DNA, the heads of the Smc2 and Smc4 subunits dynamically engage with each other and with the Smc2-Smc4 hinge. Our findings show that condensin SMC dimers are able to adopt various conformations, which suggests that condensin complexes have the structural flexibility required to engage and link the chromatin fibers of eukaryotic genomes.

## Results

### Smc2-Smc4 Dimers Display a Variety of Conformations

While this paper focuses on the results from liquid AFM, we first, as a point of reference and for comparison to reported data, used rotary shadowing EM to image Smc2-Smc4 dimers purified from budding yeast *Saccharomyces cerevisiae* (Figure 1B). Surprisingly, in only about one-third of the Smc2-Smc4 dimers, the coiled coils were closely juxtaposed over part or all of their lengths, resulting in the I- or Y-shaped conformations that were predicted from previous studies (Anderson et al., 2002, Soh et al., 2015, Yoshimura et al., 2002). The majority of molecules displayed instead clearly separated coiled coils, resulting in either V-shaped or O-shaped conformations (Figure S1A). In parallel to rotary shadowing EM, we also imaged the molecules by dry AFM (Figure 1C). Consistent with the EM data, the vast majority of molecules identified in the AFM images had separated coiled coils and appeared as V- or O-shaped dimers (Figure S1B). Notably, we never observed the coiled coils as juxtaposed stiff rods with dry AFM.

Since earlier EM and AFM studies investigated Smc2-Smc4 dimers of vertebrate and fission yeast condensin complexes, it is conceivable that the *S. cerevisiae* Smc2-Smc4 dimer represents an unusual exception to the previously reported rod-shaped architecture. We therefore purified and imaged another Smc2-Smc4 dimer, this time from the thermophilic yeast species *Chaetomium thermophilum* (Figure S1C). Similar to what we had observed for the *S. cerevisiae* Smc2-Smc4 dimer, we again found that the majority of the molecules were in either the V- or O-shaped conformations. We therefore conclude that the Smc2-Smc4 dimers of two yeast species, which diverged several hundred million years ago, can adopt a number of different conformations, with the majority in O or V shapes.

Since EM and dry AFM can only gather snapshots of protein conformations, we used high-speed AFM in liquid to create movies of *S. cerevisiae* Smc2-Smc4 dimers with a frame rate of ten frames per second. We classified the conformations of the dimers in over 1,700 frames taken with high-speed AFM (Figure 1D). The V-shaped conformation accounts for a quarter of the cases (Figure 1D, first row), while the most abundant configuration is the O-shaped conformation (Figure 1D, second row). Unexpectedly, liquid AFM imaging uncovered two additional conformations, which involve interactions between the head and hinge domains. In a conformation that we refer to as “butterfly” (B-shaped), both ATPase heads engage with the hinge and the intervening coils form two short loops that extrude from this head-hinge complex (Figure 1D, third row). In a conformation that we refer to as P-shaped conformation, only one of the heads engages with the hinge and the other head moves freely (Figure 1D, fourth row). We conclude that, in addition to the conformations also found with dry imaging techniques, high-speed AFM in liquid uncovered that Smc2-Smc4 dimers can adopt two additional conformations that had escaped prior notice.

### Smc2-Smc4 Dimers Undergo Frequent Conformational Changes

**Figure 2:**
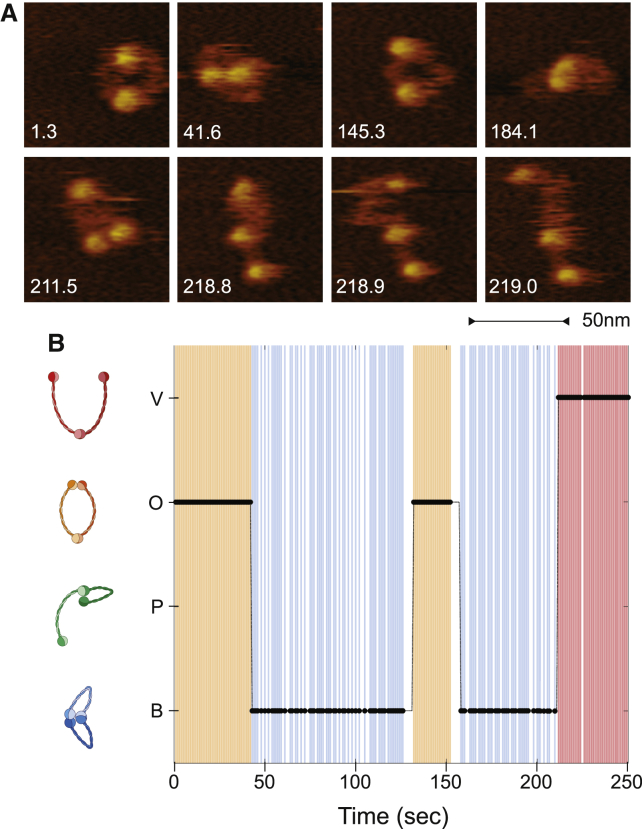
Smc2-Smc4 Dimers Change Conformations Dynamically (A) Snapshots of an Smc2-Smc4 dimer followed over time with high-speed AFM at a rate of 10 frames per second in Movie S1. Snapshots are taken at various time points in the movie (shown in seconds). (B) Annotation of conformational classes for each frame of Movie S1. O-shaped conformations are indicated in orange, B-shaped conformations in blue, V-shaped conformations in red. White gaps indicate that the conformation could not be confidently classified for a particular frame.

Analysis of an individual Smc2-Smc4 dimer recorded in real time revealed that the dimer did not remain in one static configuration during the course of the experiment (Figure 2; Movie S1). At the start of the movie, the molecule was O-shaped (first frame in Figure 2A), and then the heads approached the hinge to form a “butterfly” structure (second frame in Figure 2A). The molecule switched between O- and B-shaped conformations multiple times before converting to a V-shaped “open” conformation toward the end of the movie (last four frames in Figure 2A). Remarkably, all Smc2-Smc4 dimers that we studied underwent conformational changes during the imaging time (Figure S2; Movies S1, S2, and S3).

### Head-Head and Head-Hinge Engagements Are Dynamic

**Figure 3:**
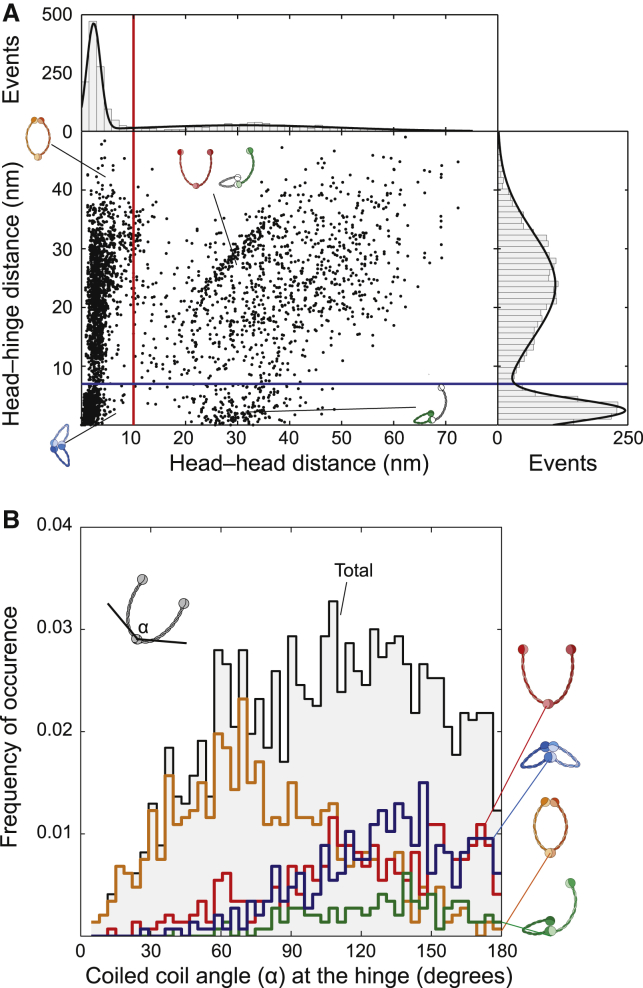
Structural Analysis of Smc2-Smc4 Conformations (A) Scatterplot of head-head distances versus head-hinge distances for each subunit of an Smc2-Smc4 dimer imaged by high-speed liquid AFM (total 1,795 frames of 18 independent molecules). Each dot represents a measurement of one SMC subunit. Frequencies of data points for head-head distances or head-hinge distances are plotted as histograms at the top and right side of the plot, respectively. Data points left of the vertical red line and below the horizontal blue line: B-shaped conformations, points left of the red and above the blue line: O-shaped conformations, points right of the red and above the blue line: V-shaped conformations and the head-hinge disengaged arm of P-shaped conformations, points right of the red line and below the blue line: the head-hinge engaged arm of P-shaped conformations. (B) Histogram plot of the angles between the Smc2-Smc4 coiled coils, measured at the hinge. The black histogram shows the frequency of occurrence for all conformations. Histograms for individual conformational classes are shown in red (V-shaped), orange (O-shaped), green (P-shaped), or blue (B-shaped).

To more carefully analyze the dynamics by which the Smc2-Smc4 ATPase heads engage with each other and with the hinge, we determined the distances between the centers of the two heads and the distances between the centers of each head to the center of the hinge. The distance between the heads (Figure 3A, top histogram) showed a clear peak, which can be fit by a Gaussian profile at 2.5 ± 1.3 nm (error denotes SD). This peak corresponds to all conformations with associated head domains, i.e., all O- and B-shaped conformations, which group on the left side of the red line in the scatterplot (Figure 3A, main panel). The head-head distance distribution also contained a second population at much larger distances of 20–60 nm (right of the red line), which corresponds to V- and P-shaped conformations. The distances between head and hinge domains (Figure 3A, right histogram) showed a large peak at 2.4 ± 1.9 nm. This peak corresponds to conformations in which at least one of the two heads engages with the hinge, i.e., all B- and P-shaped conformations in the group below the blue line in the scatterplot. The second broad peak at 23.8 ± 9.1 nm signals the large head-hinge distance in open V- and P-shaped conformations.

To quantify the degree of openness of the Smc2-Smc4 dimers, we measured the angle between the two coiled coils at the hinge. For all conformations combined (Figure 3B, black histogram), we find that the frequency of occurrence increases approximately linearly up to ∼70 degrees and then levels off for higher angles. Low angles are strikingly absent, which reflects the fact that we never observed a conformation in which the coiled coils are clamped together into a rod. Furthermore, the angle distribution depends on the conformation of the dimer. In the O-shaped conformation, the frequency of occurrence has a broad asymmetric peak with a maximum near 70 degrees. In all other conformations, we observed almost exclusively large-angle conformations.

For comparison, we also measured the hinge angles of *S. cerevisiae* Smc2-Smc4 dimers in electron micrographs (Figure S3A). We again observed a wide distribution of angles between the two coils, with a peak at around 40 degrees and a lower occurrence of smaller angles. The quantitative difference between peak values measured by EM and liquid AFM implies that vacuum drying SMC dimers on mica surfaces, an unavoidable protocol for EM, may impact the coiled coil arrangement. To exclude the possibility that the attachment of the head domains to the surface artificially biases the coiled coils into an open conformation during preparation for EM, we also measured the hinge angles of “head-less” *C. thermophilum* Smc2-Smc4 dimers in electron micrographs (Figures S3B and S3D) and compared them to the angles measured for full-length *C. thermophilum* Smc2-Smc4 dimers (Figures S1C and S3C). In both cases, we again observed a wide distribution of angles with a peak around 40 degrees. These measurements confirm that the coiled coils emanate from the Smc2-Smc4 hinge domains in an open conformation, rather than in a juxtaposed closed conformation, independent of the presence of the ATPase head domains or species origin.

### The SMC Coiled Coils Are Highly Flexible

A corollary of the finding that Smc2-Smc4 dimers can adopt a large number of conformations is that the coiled-coil structure of the SMC proteins must be very flexible and thereby allow the free movement of the head domains in relation to the hinge domain. In fact, the flexibility of the coils can be directly observed in the time-lapse recordings of the SMC dimers in liquid (Figure 2A; Movies S1, S2, and S3). Even when the molecule remains in the same conformational class, the coiled coils are highly mobile. For example, in the last three panels of the time lapse shown in Figure 2A, the Smc2–Smc4 dimer remains in the V-shaped conformation, but the coiled coils change their position between every frame (taken at 0.1-s intervals). The coils are even able to sharply bend into the B- and P-shaped conformations to enable head-hinge interactions (Figure 1D, third and forth rows), a motion that could not be achieved if the coiled coils were stiff.

**Figure 4:**
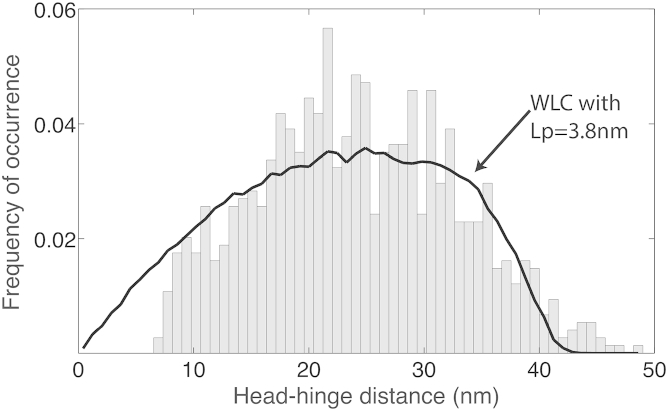
The Smc2-Smc4 Coiled Coils Can Be Characterized by a Worm-like Chain Model with a Persistence Length of ∼4 nm The measured end-to-end length histogram (head-hinge distance) of open configurations (gray bars) has a broad peak around 25 nm. This shape is well reproduced by the end-to-end distribution of 10^5^ simulated worm-like-chain polymers with a persistence length of 3.8 nm (black line).

We quantified this flexible behavior of the coiled coils by comparison to theoretical models developed for flexible polymers. The worm-like-chain (WLC) model is often used to describe the behavior of homogeneous semi-flexible polymers such as DNA or proteins (Bustamante et al., 1994, Kellermayer, 1997, van Noort et al., 2003). In the WLC model, the stiffness of a polymer is expressed as the persistence length L_P_. It can be estimated from AFM images through the mean squared end-to-end distance of the coiled coil (i.e., head-hinge distance, Rivetti et al., 1996). We took only V-shaped conformations into account (to exclude the effect of the head-hinge and head-head interactions) and fitted WLC model predictions to the histogram of end-to-end lengths of the coiled coils (Figure S4A). Because there was no closed form available to describe this distribution analytically and approximations were only published for a limited set of persistence lengths (Hamprecht et al., 2004), we generated the distributions through Monte-Carlo simulations. These simulations reproduced the histogram of head-hinge distances for V-shaped conformations quite well. We found that our data are best described by a WLC model with an L_P_ of 3.8 ± 0.2 nm and a contour length of 46 ± 2 nm (Figure 4). As a visual control, we used the same simulation algorithm and parameters to generate example shapes of dimers. The simulations strikingly resemble our observations in high-speed AFM (Figure S4B), hereby confirming our method. We conclude that the coiled coils of Smc2-Smc4 dimers can be described as flexible polymers with a persistence length of only about 4 nm.

## Discussion

Using high-speed AFM under liquid conditions, we have examined the structure and dynamics of condensin’s Smc2-Smc4 dimers in real time and under physiological conditions. Contrary to the suggestion from a crystal structure of the Smc2-Smc4 hinge domain, cross-linking experiments (Soh et al., 2015), and from images of Smc2-Smc4 dimers taken after drying them on a solid surface (Figure S1; Anderson et al., 2002, Yoshimura et al., 2002), we never observed rod-shaped molecules with their coiled coils juxtaposed under liquid conditions. We conclude that the coiled coils are not stiff rods but are instead highly flexible.

Coiled coils serve a broad range of functions in many different proteins. It is hence useful to put our finding of the highly flexible nature of the Smc2-Smc4 coiled coils (L_P_ ∼4 nm) into perspective. While a single alpha helix is very flexible (L_P_ ∼1 nm; Papadopoulos et al., 2006), coiled coils are in general significantly more stiff. Theoretically, the persistence length of a coiled coil formed by two alpha helixes has been predicted to be as high as 200 nm (Yogurtcu et al., 2010). In reality, the global stiffness of a coiled-coil structure depends on its sequence and on local interruptions by non-coiled sequences. Measured values of L_P_ of coiled-coil proteins range from 25 nm for myosin II (Schwaiger et al., 2002) to up to 100 nm for tropomyosin (Li et al., 2010, Li et al., 2012, Loong et al., 2012). All hitherto reported L_P_ values are larger than the value that we deduced for the Smc2-Smc4 dimers, emphasizing the remarkably flexible nature of these condensin subunits.

We find that Smc2-Smc4 dimers characteristically display an open structure. The discrepancy to previously reported structures can be due to several factors. First, to accurately assess the behavior of the coiled coils, it is important to take the full-length SMC proteins into account, since the engagement of the heads with each other and the hinge has an influence on the behavior of the coils. Our results indeed indicate that the heads have a certain attractive force toward each other and to the hinge. Measurements on truncated proteins that lack the heads or parts of the coiled coil could therefore yield skewed results. Second, sample preparation conditions for EM and dry AFM can result in experimental artifacts, such that certain conformations are missed. Measuring in liquid at near-physiological conditions is closer to the in vivo situation. Third, our liquid AFM data show that the configuration of the SMC dimers is dynamic over time. Consequently, results from bulk cross-linking experiments should be treated with caution, as transient interactions can be kinetically trapped with this method. Moreover, the open conformations would be missed and cross-linking interactions might occur between adjacent molecules.

We find that the ATPase heads of the Smc2-Smc4 dimers engage and disengage with each other in a dynamic manner. Excitingly, we find that the heads also interact dynamically with the hinge, resulting in a hitherto undiscovered structure. The dip in the head-hinge distance probability density at ∼7 nm (Figure 3A, right panel) suggests that this interaction takes place despite a considerable entropic penalty. In the P-shaped form, only one head interacts with the hinge, which indicates that the two ATPase heads interact with the hinge independently of each other. It has previously been suggested that the head and hinge domains of cohesin’s Smc1–Smc3 dimer might need to associate to enable an ATP hydrolysis-driven disengagement of the hinge for DNA entry into the cohesin ring, a feat that can only be achieved by the folding of the intervening coiled coils (Gruber et al., 2006, Nasmyth, 2011). If DNA enters condensin rings in an analogous manner to what has been proposed for cohesin, then the newly identified “butterfly” structure reveals a conformation that is important in condensin’s DNA loading mechanism.

Here, we demonstrated that high-speed AFM in liquid is able to provide a quantitative analysis of the dynamics of SMC dimers under physiological conditions. We showed that even in the absence of ATP or DNA, Smc2-Smc4 dimers adopt highly dynamic and flexible conformations. The biophysical properties of the SMC coiled coils revealed by our study provide the fundamental basis for the mechanics of DNA entrapment by the SMC protein machinery and set the stage for further in-depth biochemical and structural studies of condensin and cohesin.

## Experimental Procedures

### Purification of Smc2-Smc4 Dimers

*S. cerevisiae* Smc2 fused to a C-terminal His_6_ epitope tag and Smc4 fused to a C-terminal StrepII tag were co-expressed from an episomal plasmid under the control of the galactose-inducible *GAL1* or *GAL10* promoters in protease-deficient budding yeast cells (strain C2598) and purified via Ni-NTA, StrepTactin and gel filtration steps as described (Piazza et al., 2014).

### EM and Rotary Shadowing

Smc2–Smc4 protein preparations were dialyzed for 45 min against 200 mM NH_4_HCO_3_, 30% glycerol, and 2 mM DTT (pH 7.6). 3 μl of 0.1 mg/ml dialyzed Smc2-Smc4 dimers was sprayed onto freshly cleaved mica, immediately dried in vacuum, and rotary shadowed with Pt-C at an angle of 7°. Images were recorded in a Morgagni FEI microscope at 56,000× magnification.

### Dry AFM

SMC dimers were diluted to a concentration of 7.1 μg/ml in 200 mM NaCl, 10 mM Tris-HCl (pH 7.0), 30 mM MgCl_2_, 5 mM DTT, and 10% glycerol. Samples were incubated on mica for 10 s before rinsing with MilliQ water and drying with a nitrogen gun. Imaging was performed on a Bruker Multimode AFM, using BudgetSensors SHR150 ultrasharp probes.

### High-Speed AFM in Liquid

Purified Smc2-Smc4 dimers at a concentration of 2.2 mg/ml were 20× diluted with imaging buffer (20 mM Tris-HCl [pH 7.0], 200 mM NaCl, 10 mM MgCl_2_, 5 mM DTT, 10% glycerol) and immediately snap-frozen in aliquots and stored at –80°C. Prior to imaging, samples were thawed and diluted another 40× with imaging buffer, and a droplet of protein solution was applied to freshly cleaved mica. After 10 s, the surface was rinsed with imaging buffer and placed—without drying—into the imaging bath of the AFM (HS-AFM 1.0, RIBM). Procedures for imaging were largely according to those described in published protocols (Uchihashi et al., 2012). Nanoworld USC-f1.2-k0.15 and USC-f1.5-k-0.6 cantilevers were used. AFM movies of selected areas with single molecules, typically 70 × 80 nm in size, were acquired at frame rates of 2–10 Hz. The tip forces are controlled through a system that stabilizes the oscillation via a feedback mechanism on the second harmonic amplitude (Kodera et al., 2006, Schiener et al., 2004).

### Image Analysis

A user-guided semi-automatic image analysis was performed. Because of the large data volume, only every fifth frame of the AFM movies was analyzed. This resulted in a total of more than 1,700 data points.

### Stiffness Analysis and Monte Carlo Simulations

We simulated two-dimensional worm-like chains by dividing each chain into N segments of length L_s_ = 0.2 nm, and assigning angles α between the segments that are drawn from a normal distribution with a variance L_p_/L_s_. This definition of the persistence length L_p_ follows the analysis of Rivetti et al. (1996). From the angles between segments, x and y coordinates are calculated for the entire chain. For each value of the persistence length and contour length, 10^6^ chains are simulated (see Figure S4B for examples), and histograms are calculated of the end-to-end distances. Using the sum of the least-squares between the Monte Carlo results and the experimental data as a goodness-of-fit estimator, the values of L_p_ and L_c_ are iterated to obtain the best fits and confidence intervals. The errors of the fits are one SD confidence intervals, obtained through the graphical Monte Carlo method (Cowan, 1998). We note that this procedure significantly extends beyond the traditional approach first described by Rivetti et al., which yields an estimate for the persistence length using only the mean square end-to-end distance. In our case, that approach yields a value of 4.6 nm. The difference can be ascribed to the fact that data for end-to-end distances near zero are missing from the distribution due to the head-hinge interactions. Finally, it should be noted that in our experimental data, the centers of the heads and hinges are taken as markers for the ends of the coiled coils; i.e., we do not take the finite size of the hinge and head domains into account, nor the (unknown) position of the attachment points.

## Author Contributions

Conceptualization, J.M.E., A.J.K., C.H.H., and C.D.; Formal Analysis, J.M.E., A.J.K., and C.H.H.; Software, J.M.E. and A.J.K.; Investigation, A.J.K., E.M.D., L.d.W., M.K., M.H., and C.H.H.; Writing, J.M.E., A.J.K., C.H.H., and C.D.; Supervision, C.H.H. and C.D.

## Supplemental Information

Document S1. Supplemental Experimental Procedures, Figures S1–S4, and Table S1

Movie S1. Movie of an Smc2-Smc4 Dimer Taken with High-Speed AFM, Related to Figure 2 This movie corresponds to the time trace in Figure 2B.

Movie S2. Movie of an Smc2-Smc4 Dimer Taken with High-Speed AFM, Related to Figure 2 This movie corresponds to the time trace in Figure S2F.

Movie S3. Movie of an Smc2-Smc4 Dimer Taken with High-Speed AFM, Related to Figure 2 This movie corresponds to the time trace in Figure S2G.

Document S2. Article plus Supplemental Information
