# Supplementary material for: Condensin Smc2-Smc4 Dimers Are Flexible and Dynamic
Source: Cell Rep. 2016 Feb 18;14(8):1813–8. doi: 10.1016/j.celrep.2016.01.063 (PMC4785793; doi:10.1016/j.celrep.2016.01.063)
Supplement: Document S1. Supplemental Experimental Procedures, Figures S1–S4, and Table S1 [file mmc1.pdf]

Cell Reports, Volume *14*

Supplemental Information

Condensin Smc2-Smc4 Dimers Are Flexible and Dynamic

Jorine M. Eeftens, Allard J. Katan, Marc Kschonsak, Markus Hassler, Liza de Wilde, Essam M. Dief, Christian H. Haering, and Cees Dekker

Figure S1

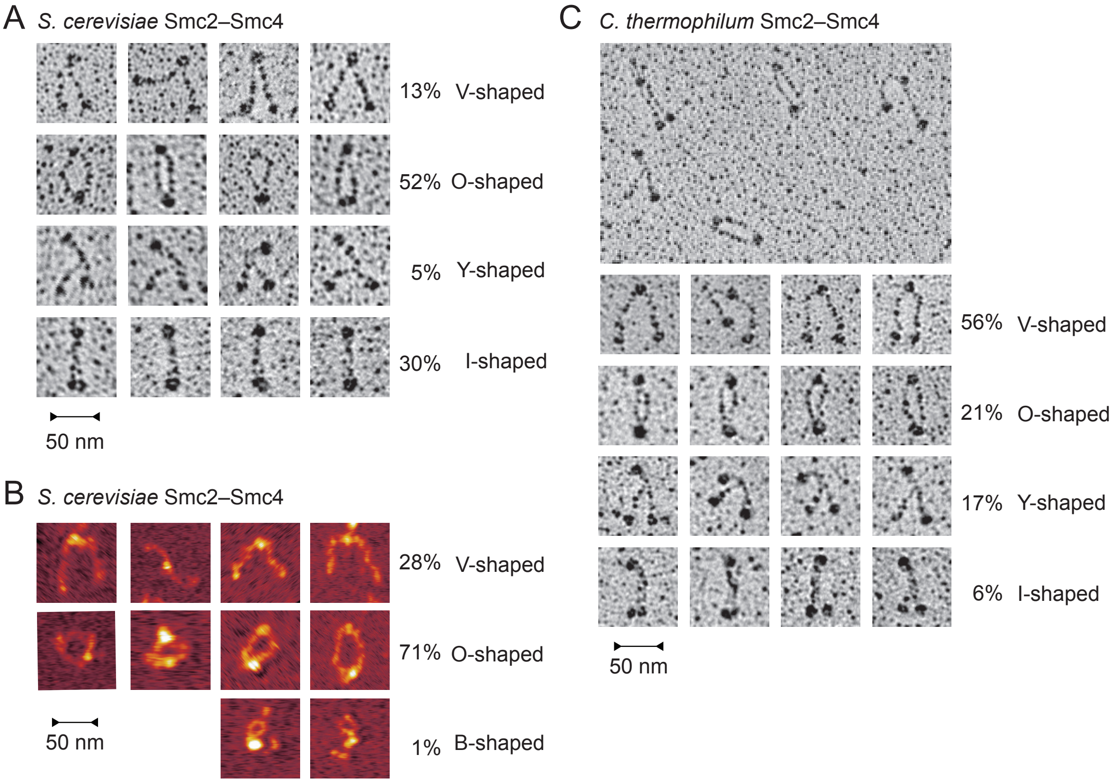

Figure S1: Smc2–Smc4 dimer conformations in EM and dry AFM, related to Figure 1

(A) Examples of different conformation classes of *S. cerevisiae* Smc2–Smc4 dimers observed by rotary shadowing EM. Frequencies of occurrence are listed on the right as a fraction of 500 molecules classified. Molecules were categorized as O-shaped if a space of at least the width of a coiled coil was visible between the SMC coiled coils. **(B)** Examples of different conformations of *S. cerevisiae* Smc2–Smc4 dimers observed by dry AFM. Frequencies of occurrence are listed on the right as a fraction of 400 molecules classified. **(C)** Examples of different conformations of *Ch. thermophilum* Smc2–Smc4 dimers observed by rotary shadowing EM. Frequencies of occurrence are listed on the right as a fraction of 400 molecules classified.

Figure S2

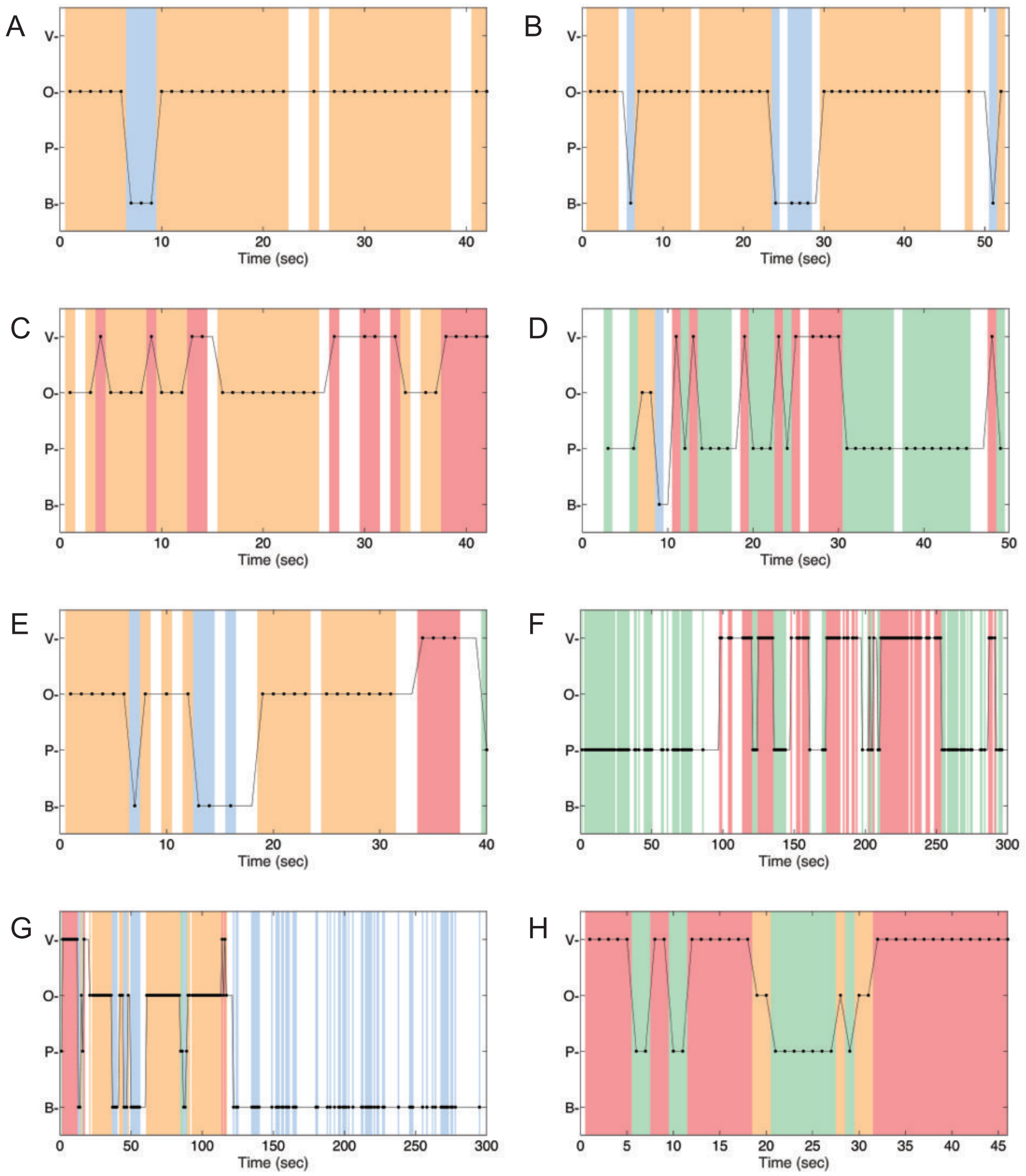

Figure S2: Conformational changes of different Smc2–Smc4 dimers followed by high-speed AFM, related to Figure 2

Conformations of individual frames of eight movies of different Smc2–Smc4 molecules were classified. V-shaped in red, O-shaped in orange, P-shaped in green, B-shaped in blue. White gaps indicate that the conformation could not be confidently classified for a particular frame.

Figure S3

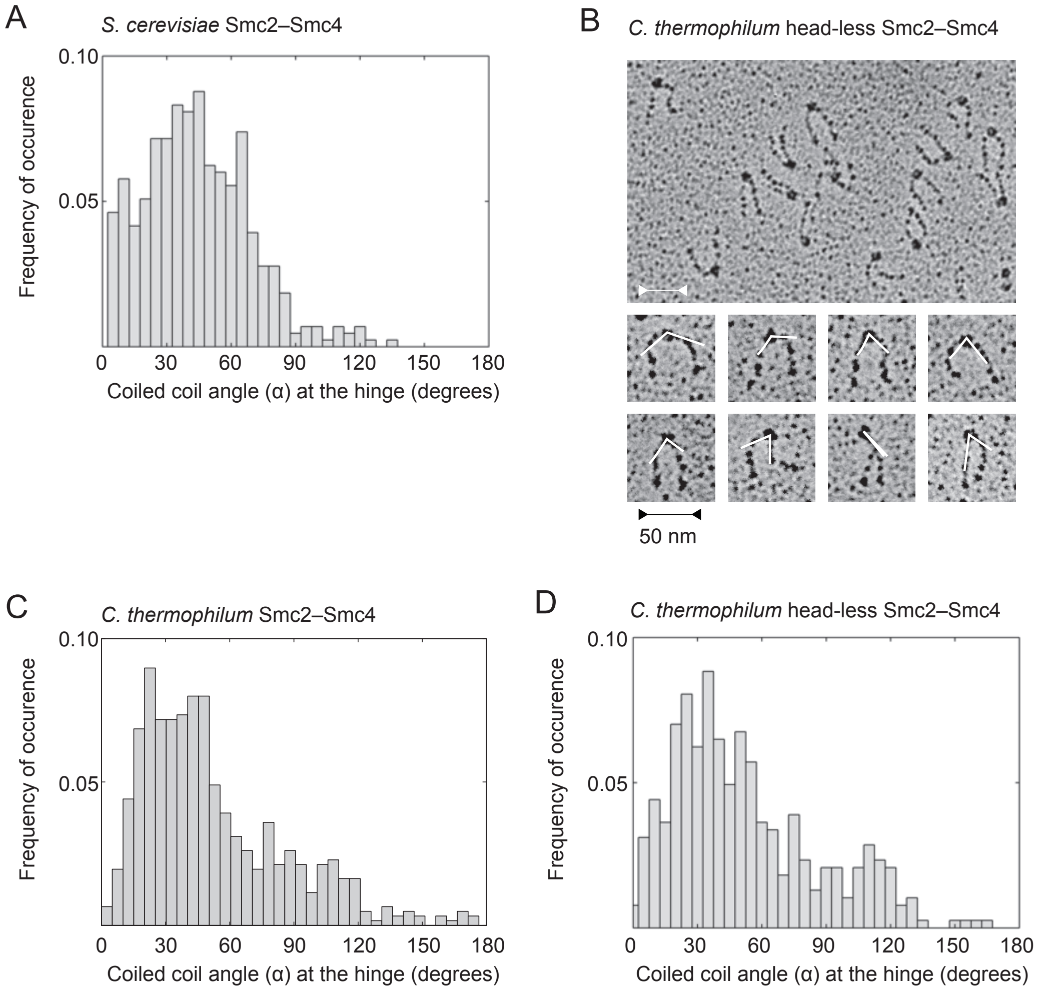

Figure S3: Coiled coil angle measured in electron micrographs, related to Figure 3

(A) Histogram frequency plot of coiled-coil angles measured at the hinge of full-length *S. cerevisiae* Smc2–Smc4 dimers (displayed as fraction of a total 430 individual molecules). **(B)** Example image of ‘head-less *Ch. thermophilum* Smc2-Smc4 dimers imaged by rotary shadowing EM. **(C)** Histogram frequency plot of the coiled coil angles at the hinge of head-less *Ch. Thermophilum* Smc2–Smc4 dimers (displayed as fraction of a total 385 individual molecules). **(D)** Histogram frequency plot of the coiled coil angles at the hinge *Ch. Thermophilum* Smc2–Smc4 dimers (displayed as fraction of a total 613 individual molecules).

Figure S4

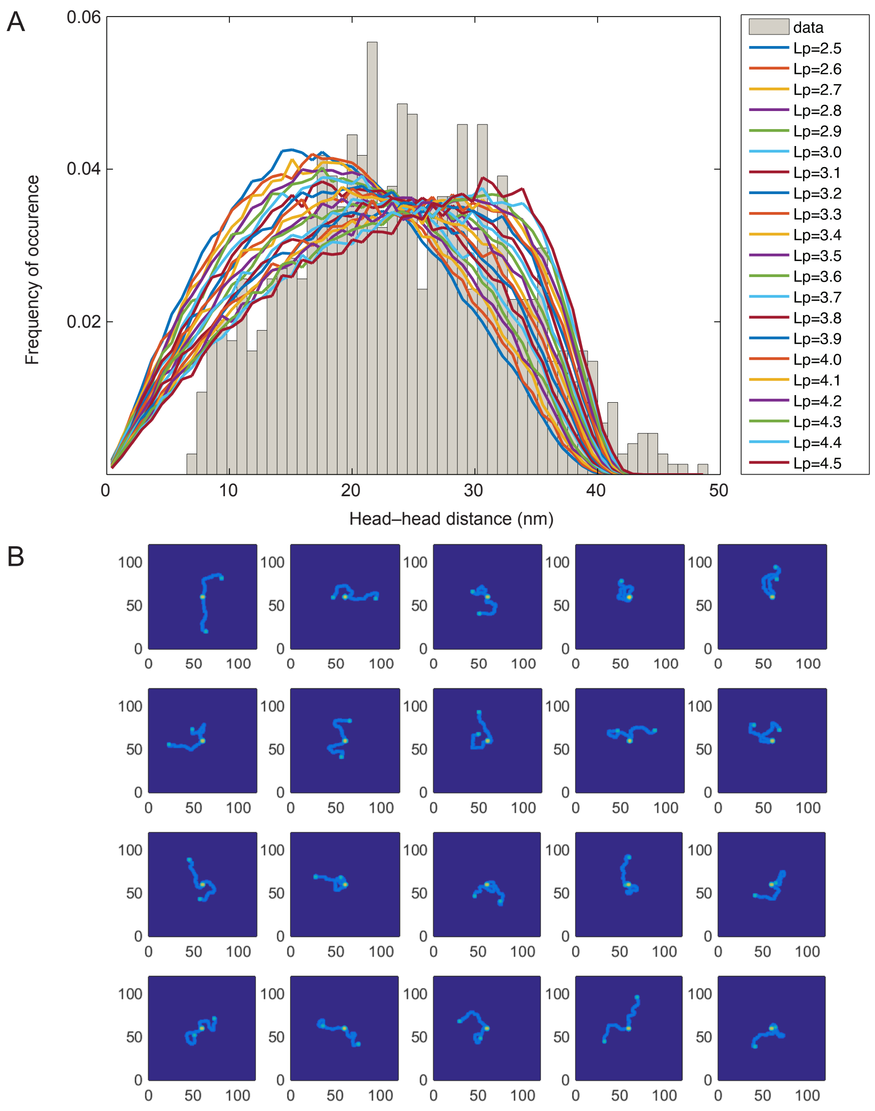

Figure S4: A worm-like chain model to describe the behavior of Smc2–Smc4 dimers, related to Figure 4

(A) We simulated worm-like chains with varying persistence length and a set contour length of 46 nm. A persistence length of 3.8 nm fits our data measured for *S. cerevisiae* Smc2–Smc4 dimers in the V-shaped (grey histogram) best, as judged from minimizing for the least squared residues. **(B)** We used the found persistence length of 3.8 nm to simulate the behavior of the coiled-coils. The results of these simulations resemble our AFM images.

Table S1:

Persistence lengths of coiled coils and other biological polymers, ranged from stiff to flexible, related to Figure 4.

**Polymer**	**Persistence length**	**Reference**
Coiled coil (theorerical)	200nm	Yogurtcu et al., 2010
Tropomyosin coiled coil	40-100nm	Li et al., 2010, 2012; Loong et al., 2012
Vimentin coiled coil	63nm	Ackbarow and Buehler, 2007
Double stranded RNA	62nm	Abels et al., 2005
Double stranded DNA	50nm	Bustamante et al., 1994
Rad50 coiled coil	35nm	de Jager et al., 2001
Myosin II coiled coil	25nm	Schwaiger et al., 2002
Smc2-Smc4 coiled coil	*4nm*	*this manuscript*
Protein alpha helix	1nm	Papadopoulos et al., 2006
Single strand DNA	0.8nm	Smith et al., 1996
Unstructured amino acid chain	0.4nm	Carrion-Vazquez et al., 1999

Supplemental Movies

Movie S1: Movie of an Smc2-Smc4 dimer taken with high-speed AFM, related to Figure 2.

This movie corresponds to the time trace in Figure 2B.

Movie S2: Movie of an Smc2–Smc4 dimer taken with high-speed AFM, related to Figure 2.

This movie corresponds to the time trace in Figure S2F.

Movie S3: Movie of an Smc2-Smc4 dimer taken with high-speed AFM, related to Figure 2.

This movie corresponds to the time trace in Figure S2G.

Supplemental experimental procedures

Force control in AFM imaging and the possible influence of the scanning motion on SMC dynamics

We operate the AFM in intermittent contact ('tapping') mode. The tapping amplitude A during imaging is approximately 4 nm, and the 'amplitude ratio' (imaging amplitude divided by free amplitude) is typically 85-95%. The imaging feedback keeps the amplitude A constant, but because the drive efficiency of the tapping piezo is subject to temporal fluctuations, the amplitude ratio can fluctuate substantially outside the user's control. To have more control over the applied forces, A_2, the second harmonic of the cantilever's sinusoidal motion is monitored using a Signal Recovery model 7280 lock-in amplifier and kept constant by a homebuilt feedback unit, that modulates the drive amplitude. In the range of imaging parameters that we use, A_2 is monotonic in the strength of the tip-sample interaction, and therefore it is a good proxy for the imaging force. However, the exact relation between A_2 and force depends on several unknown parameters and is therefore not quantifiable.

The bandwidth of the AFM imaging system depends on the force that is applied to the sample. At the lowest imaging forces, the image quality is poor and only the location, but not the conformation of the dimers can be seen. Evaluation of these images indicates that the mobility of the dimers is not reduced under these imaging conditions compared to the higher forces used to obtain quantitative results, suggesting that the tip influence on these results is minimal. To obtain images that are sharp enough to distinguish all four configurations from each other while maintaining an image speed of 2 frames/s, we find that an A_2 value of 20 pm or more is required. Values of 50-70 pm are sufficient to obtain high-quality image*s* up to 10 frames/s, and hence we did not go beyond this. Notably, within the range of forces that yields movies of sufficient quality, we find no effect of the imaging on the dynamics of the SMC dimers. In addition, we see no correlation between the head positions and the fast scanning direction. We therefore conclude that there is no indication that the AFM scanning induces changes to the molecular configuration of the SMC dimers or the dynamic behavior of the heads through energy input from the tip.

Purification of full-length Chaetomium thermophilum Smc2-Smc4 dimers

C. thermophilum Smc2 fused to a C-terminal StrepII tag and Smc4 (residues 1-1,542) fused to a C-terminal His_8 tag were co-expressed in Sf21 cells cultured in Sf-900 III SFM serum-free medium (Invitrogen) from a single baculovirus. About $0.5\text{-}0.8\times10^8$ Sf21 cells were lysed by sonication in lysis buffer (50mM TRIS-HCl pH 7.5, 300mM NaCl, 20mM imidazole, 5mM β-mercaptoethanol) supplemented with 1× Complete EDTA-free protease inhibitor cocktail (Roche) at 4˚C. The lysate was cleared by centrifugation at 45,000×g for 50min at 4˚C and incubated with NiNTA Sepharose (GE Healthcare) for 2h at 4˚C. The resin was washed with ~100 column volumes (CV) lysis buffer before elution with 4 CV elution buffer (50mM TRIS-

HCl pH 7.5, 300mM NaCl, 320mM imidazole, 5mM β-mercaptoethanol). Eluted protein fractions were combined and dialyzed over night at 4˚C in dialysis buffer (25mM TRIS-HCl pH 7.5, 300mM NaCl, 1mM DTT), diluted with 10 volumes of low-salt buffer (25mM TRIS-HCl pH 7.5, 100mM NaCl, 1mM DTT), and loaded onto a 6ml-RESOURCE Q anion-exchange column (GE Healthcare). The resin was washed with 5 CV low-salt buffer and eluted in a 60ml linear gradient to high-salt buffer (25mM TRIS-HCl pH 7.5, 1 M NaCl, 1mM DTT). Peak fractions were collected and concentrated by ultrafiltration with a 100,000 MWCO cut-off Vivaspin concentrator (Sartorius).

Purification of head-less Chetomium Thermophilum Smc2-Smc4 dimers

N-terminally His_6-tagged Smc2 (residues 224-982) and Smc4 (residues 509-1,258) from *C. thermophilum* were co-expressed in *E. coli* strain BL21(DE3)Rosetta2 pLysS (Novagen) grown in 2×TY medium and induced with 200μM IPTG at 18°C for 16h. Cell pellets were resuspended in lysis buffer (50mM TRIS-HCl pH 7.5, 500mM NaCl, 25 mM imidazole, 5mM β-mercaptoethanol) supplemented with 1× Complete EDTA-free protease inhibitors and lysed using a sonicator (Branson). Cell debris were pelleted for 1h at 40,000×g and the supernatant was incubated with NiNTA Sepharose (GE Healthcare) pre-equilibrated in lysis buffer. The resin was extensively washed with lysis buffer and eluted with 250mM imidazole. Eluted protein was dialyzed to 50mM TRIS-HCl pH 7.5, 100mM NaCl, 1mm DTT and then loaded onto a RESOURCE Q anion-exchange column equilibrated in the same buffer. Proteins were eluted with a linear gradient to 1M NaCl. The single peak fraction was concentrated and passed over a Superdex S200 26/60 gel filtration column (GE Healthcare) in 25mM TRIS pH 7.5, 200mM NaCl, 1mM DTT, where the proteins eluted in a single peak. Peak fractions were concentrated using a Vivaspin concentrator, flash frozen in liquid nitrogen, and stored at -80°C.

Supplemental references

Abels, J.A., Moreno-Herrero, F., van der Heijden, T., Dekker, C., and Dekker, N.H. (2005). Single-molecule measurements of the persistence length of double-stranded RNA. Biophys. J. *88*, 2737–2744.

Ackbarow, T., and Buehler, M.J. (2007). Superelasticity, energy dissipation and strain hardening of vimentin coiled-coil intermediate filaments: atomistic and continuum studies. J. Mater. Sci. *42*, 8771–8787.

Brown André, E.X., Litvinov, R.I., Discher, D.E., and Weisel, J.W. (2007). Forced Unfolding of Coiled-Coils in Fibrinogen by Single-Molecule AFM. Biophys. J. *92*, L39–L41.

Bustamante, C., Marko, J.F., Siggia, E.D., and Smith, S. (1994). Entropic elasticity of lambda-phage DNA. Science *265*, 1599–1600.

Carrion-Vazquez, M., Oberhauser, A.F., Fowler, S.B., Marszalek, P.E., Broedel, S.E., Clarke, J., and Fernandez, J.M. (1999). Mechanical and chemical unfolding of a single protein: A comparison. Proc. Natl. Acad. Sci. *96*, 3694–3699.

de Jager, M., van Noort, J., van Gent, D.C., Dekker, C., Kanaar, R., and Wyman, C. (2001). Human Rad50/Mre11 Is a Flexible Complex that Can Tether DNA Ends. Mol. Cell *8*, 1129–1135.

Li, X.E., Holmes, K.C., Lehman, W., Jung, H., and Fischer, S. (2010). The shape and flexibility of tropomyosin coiled coils: implications for actin filament assembly and regulation. J. Mol. Biol. *395*, 327–339.

Li, X.E., Suphamungmee, W., Janco, M., Geeves, M.A., Marston, S.B., Fischer, S., and Lehman, W. (2012). The flexibility of two tropomyosin mutants, D175N and E180G, that cause hypertrophic cardiomyopathy. Biochem. Biophys. Res. Commun. *424*, 493–496.

Loong, C.K.P., Zhou, H.-X., and Chase, P.B. (2012). Persistence length of human cardiac α-tropomyosin measured by single molecule direct probe microscopy. PLoS One *7*, e39676.

Papadopoulos, P., Floudas, G., Schnell, I., Lieberwirth, I., Nguyen, T.Q., and Klok, H.-A. (2006). Thermodynamic confinement and alpha-helix persistence length in poly(gamma-benzyl-L-glutamate)-b-poly(dimethyl siloxane)-b-poly(gamma-benzyl-L-glutamate) triblock copolymers. Biomacromolecules *7*, 618–626.

Rief, M., Pascual, J., Saraste, M., and Gaub, H.E. (1999). Single molecule force spectroscopy of spectrin repeats: low unfolding forces in helix bundles. J. Mol. Biol. *286*, 553–561.

Smith, S.B., Cui, Y., and Bustamante, C. (1996). Overstretching B-DNA: The Elastic Response of Individual Double-Stranded and Single-Stranded DNA Molecules. Science (80-.). *271*, 795–799.
