# Supplementary figures and images for: Condensin Smc2-Smc4 Dimers Are Flexible and Dynamic
Source: Cell Rep. 2016 Feb 18;14(8):1813–8. doi: 10.1016/j.celrep.2016.01.063 (PMC4785793; doi:10.1016/j.celrep.2016.01.063)

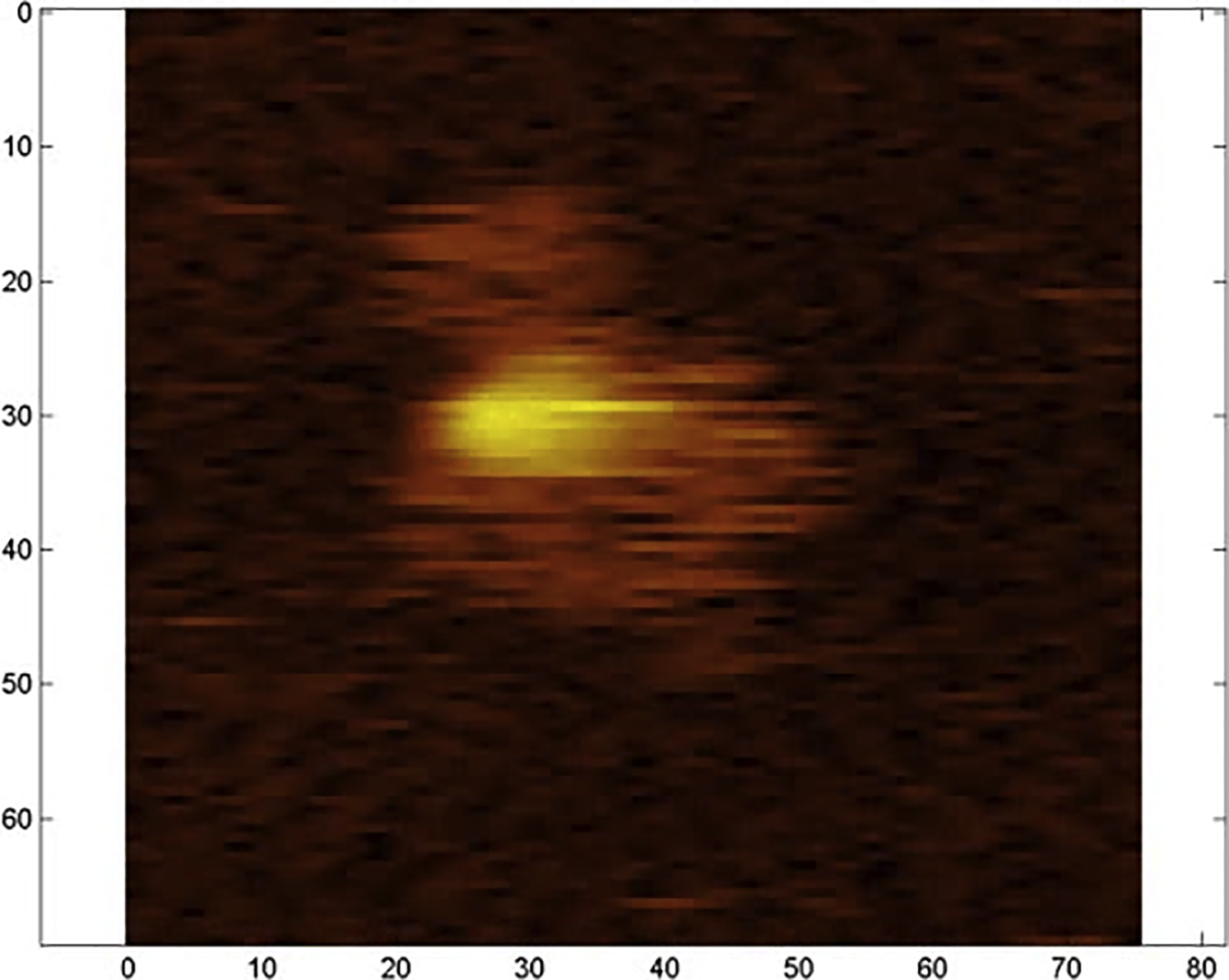

Supplement: Movie S1. Movie of an Smc2-Smc4 Dimer Taken with High-Speed AFM, Related to Figure 2 — This movie corresponds to the time trace in Figure 2B. [file mmc2.jpg]

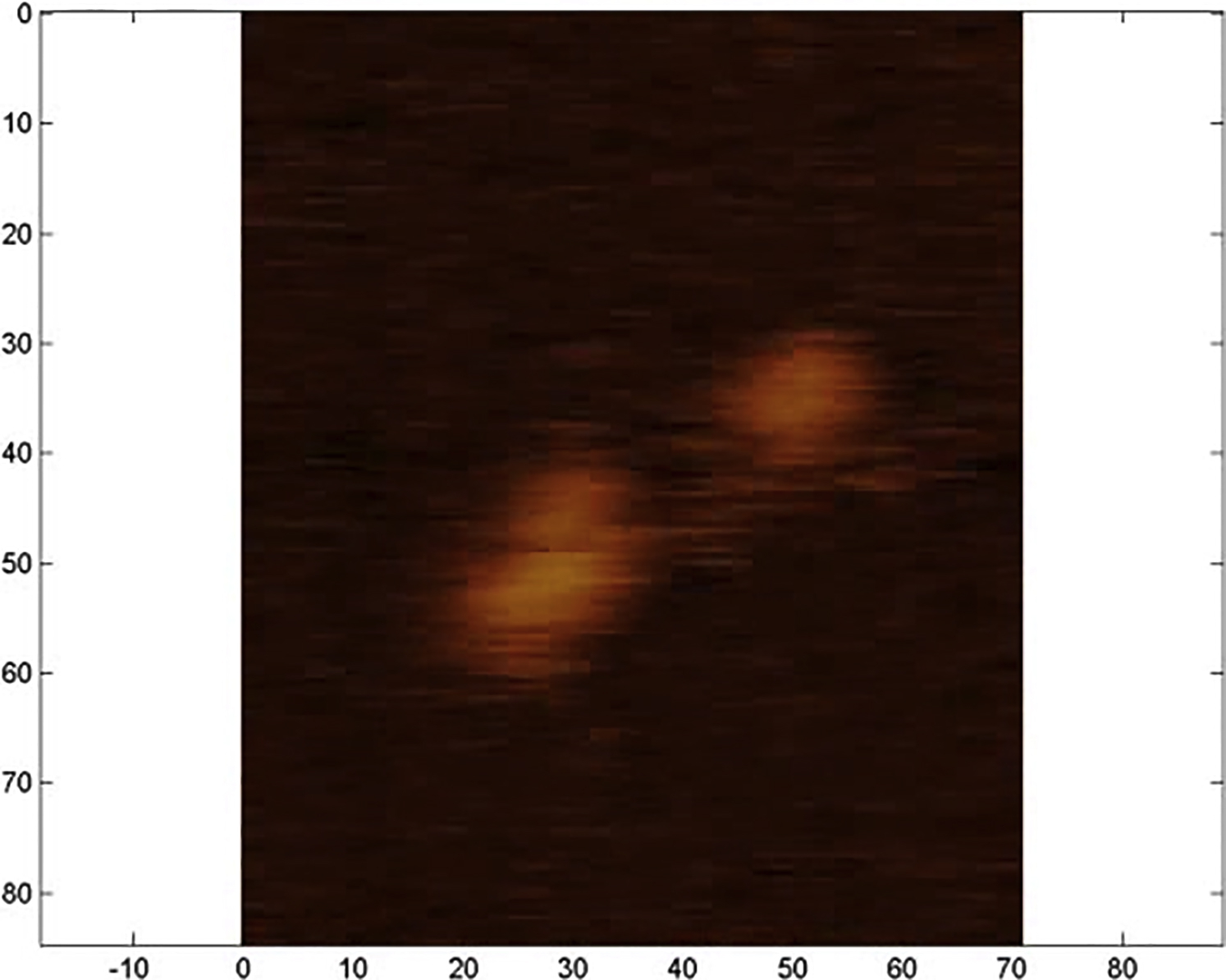

Supplement: Movie S2. Movie of an Smc2-Smc4 Dimer Taken with High-Speed AFM, Related to Figure 2 — This movie corresponds to the time trace in Figure S2F. [file mmc3.jpg]

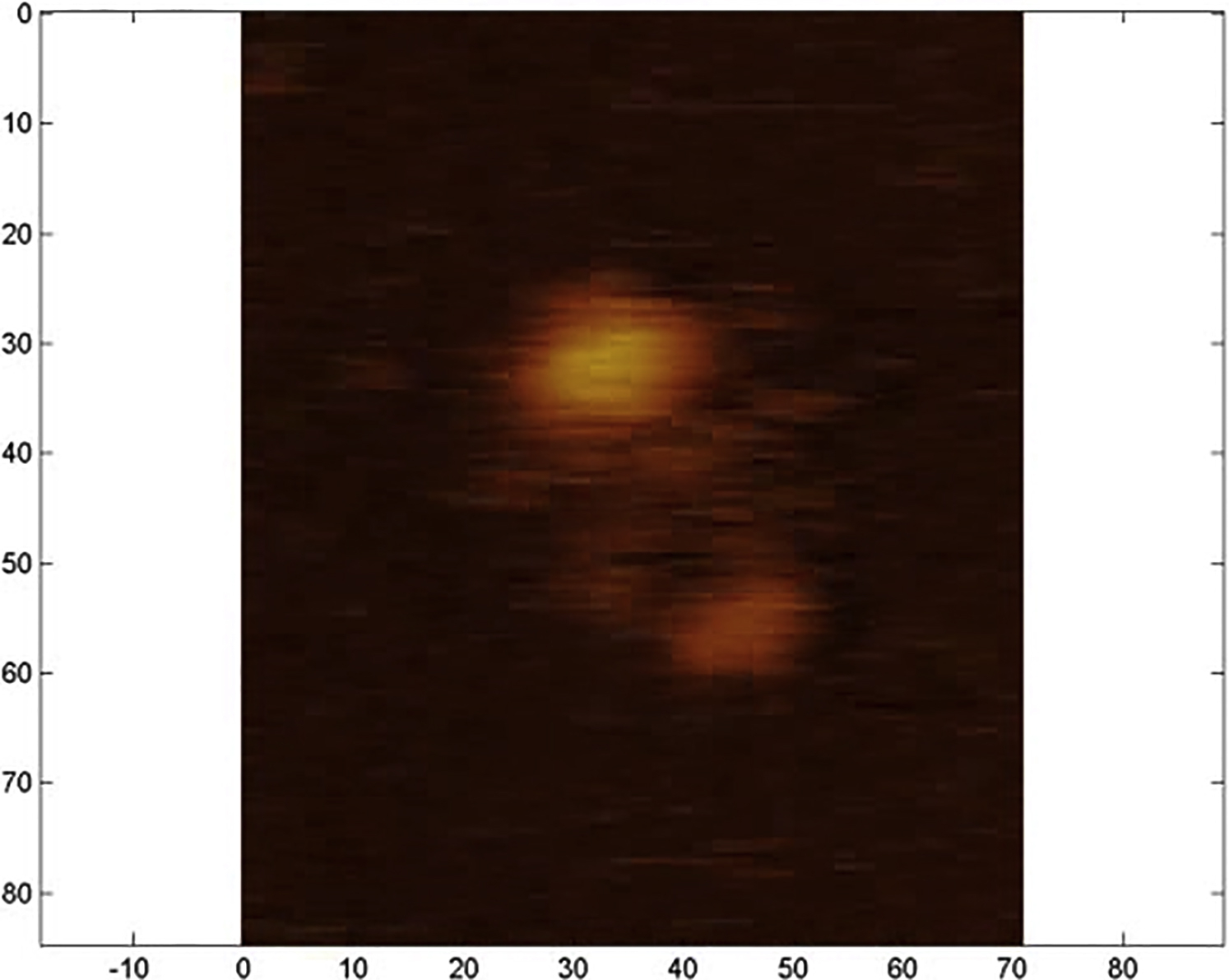

Supplement: Movie S3. Movie of an Smc2-Smc4 Dimer Taken with High-Speed AFM, Related to Figure 2 — This movie corresponds to the time trace in Figure S2G. [file mmc4.jpg]
