# Supplementary material for: Condensin Smc2-Smc4 Dimers Are Flexible and Dynamic
Source: Cell Rep. 2016 Feb 18;14(8):1813–8. doi: 10.1016/j.celrep.2016.01.063 (PMC4785793; doi:10.1016/j.celrep.2016.01.063)
Supplement: Document S2. Article plus Supplemental Information [file mmc5.pdf]

Cell Reports

Report

Condensin Smc2-Smc4 Dimers Are Flexible and Dynamic

Graphical Abstract

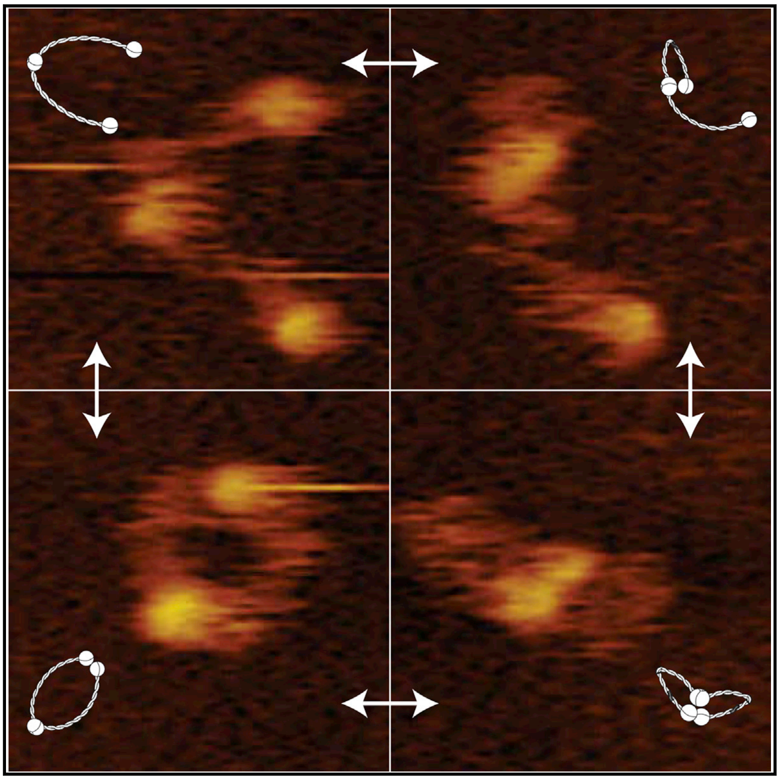

Highlights

- The conformation and dynamics of SMC dimers were imaged with high-speed AFM
- The heads of Smc2 and Smc4 engage with each other and with the hinge dynamically
- The coiled coils of Smc2 and Smc4 are flexible and show extensive fluctuations in time

Authors

Jorine M. Eeftens, Allard J. Katan, Marc Kschonsak, ..., Essam M. Dief, Christian H. Haering, Cees Dekker

Correspondence

christian.haering@embl.de (C.H.H.), c.dekker@tudelft.nl (C.D.)

In Brief

Eeftens et al. probe the topology and dynamics of condensin's Smc2-Smc4 dimers with high-speed AFM in liquid. They find that the Smc2-Smc4 coiled coils are remarkably flexible and do adopt various conformations that interconvert dynamically over time.

Eeftens et al., 2016, Cell Reports *14*, 1813–1818
March 1, 2016 ©2016 The Authors
http://dx.doi.org/10.1016/j.celrep.2016.01.063

Cell Reports
Report

OPEN ACCESS
CellPress

Condensin Smc2-Smc4 Dimers Are Flexible and Dynamic

Jorine M. Eeftens,[1,3] Allard J. Katan,[1,3] Marc Kschonsak,[2] Markus Hassler,[2] Liza de Wilde,[1] Essam M. Dief,[1] Christian H. Haering,[2,*] and Cees Dekker[1,*]
[1]Department of Bionanoscience, Kavli Institute of Nanoscience Delft, Delft University of Technology, Delft 2628 CJ, the Netherlands
[2]Cell Biology and Biophysics Unit, European Molecular Biology Laboratory (EMBL), 69117 Heidelberg, Germany
[3]Co-first author
*Correspondence: christian.haering@embl.de (C.H.H.), c.dekker@tudelft.nl (C.D.)
http://dx.doi.org/10.1016/j.celrep.2016.01.063
This is an open access article under the CC BY license (http://creativecommons.org/licenses/by/4.0/).

SUMMARY

Structural maintenance of chromosomes (SMC) protein complexes, including cohesin and condensin, play key roles in the regulation of higher-order chromosome organization. Even though SMC proteins are thought to mechanistically determine the function of the complexes, their native conformations and dynamics have remained unclear. Here, we probe the topology of Smc2-Smc4 dimers of the *S. cerevisiae* condensin complex with high-speed atomic force microscopy (AFM) in liquid. We show that the Smc2-Smc4 coiled coils are highly flexible polymers with a persistence length of only ~4 nm. Moreover, we demonstrate that the SMC dimers can adopt various architectures that interconvert dynamically over time, and we find that the SMC head domains engage not only with each other, but also with the hinge domain situated at the other end of the ~45-nm-long coiled coil. Our findings reveal structural properties that provide insights into the molecular mechanics of condensin complexes.

INTRODUCTION

Cohesin and condensin protein complexes play central roles in many aspects of chromosome biology, including the segregation of sister chromatids during cell divisions, compaction of chromosomes, and regulation of gene expression during interphase (reviewed in Aragon et al., 2013; Hirano, 2006). Although functionally different, cohesin and condensin have similar architectures: both complexes are composed of two different SMC subunits and a subunit of the kleisin protein family. Together, these three proteins form a ring-like structure that is conserved from bacteria to eukaryotes. The protein chain of each SMC protein folds back onto itself to form an ~45-nm-long antiparallel coiled coil, which connects a globular "hinge" domain at one end to an ATPase "head" domain, created by the association of N- and C-terminal protein sequences, at the other end (Figure 1A). Two SMC proteins form a heterodimer by the association of their hinge domains: Smc1-Smc3 in the case of cohesin and Smc2-Smc4 in the case of condensin (Anderson et al., 2002). In addition, the head domains of the two SMC subunits can associate in the presence of ATP. The functional roles of ATP binding-mediated dimerization and hydrolysis-dependent dissociation of the two head domains have remained largely unclear. Both cohesin and condensin have been suggested to bind to chromosomes by encircling chromatin fibers topologically within their SMC-kleisin rings (Cuylen et al., 2011; Haering et al., 2008).

The conformation and dynamics of SMC dimers are of great importance, since they are thought to mechanistically determine the biological function of all SMC protein complexes. Accordingly, there have been numerous efforts to gain insight into the configuration of the SMC dimers. Electron microscopy (EM) images of cohesin complexes suggest that the Smc1–Smc3 coiled coils emerge from the hinge domain in an open conformation, resulting in V- or O-shaped arrangements with the two coils separated along most of their lengths (Anderson et al., 2002; Haering et al., 2002; Huis in 't Veld et al., 2014). V-shaped conformations were also observed for condensin's Smc2-Smc4. However, in a large fraction of molecules the Smc2-Smc4 coiled coils seemed to align, resulting in rod- or I-shaped rather than V-shaped conformations (Anderson et al., 2002; Yoshimura et al., 2002). Support to the notion that condensin's SMC coiled coils tightly associate with each other came from a recent crystal structure of the Smc2-Smc4 hinge domains and parts of the adjacent coiled coils, as well as from chemical cross-linking experiments (Barysz et al., 2015; Soh et al., 2015). Small Angle X-ray Scattering (SAXS) experiments implied that also the SMC subunits of cohesin and prokaryotic SMC complexes form I-shaped molecules in solution (Soh et al., 2015). These contradicting results indicate that it is still unclear which configurations SMC dimers adopt in vivo, and under which circumstances conformational changes might occur. The major disadvantages of all methods that have so far been used to study the configuration of SMC molecules are that they probed the protein structure either in highly artificial environments (e.g., dried in vacuum or packed into a crystal lattice) or in a kinetically trapped state (e.g., by cross-linking).

Atomic force microscopy (AFM) has proved to be a powerful tool to visualize biomolecules and to study their mechanical properties at nanometer resolution without the need for labeling. Importantly, it can also be carried out in aqueous solution under physiological conditions. Recent technical advances have made it possible to observe single molecules in action with high-speed AFM, reaching frame rates of up to 20 frames per second and thereby allowing imaging in real time (Ando et al., 2001; Katan and Dekker, 2011). Here, we use high-speed AFM in liquid, in

CrossMark

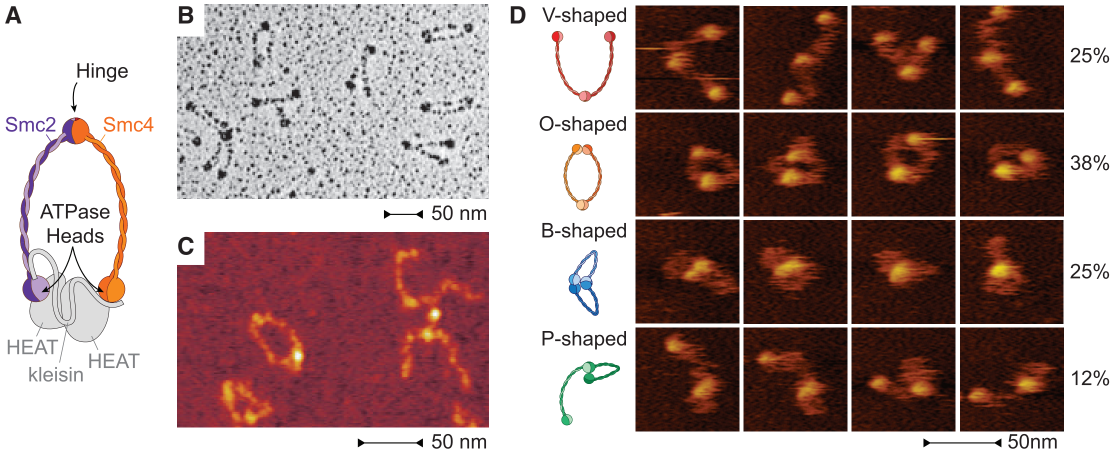

Figure 1. *S. cerevisiae* Smc2-Smc4 Dimers Adopt a Variety of Conformations

(A) Cartoon of the eukaryotic condensin complex. Smc2 and Smc4 heterodimerize via their hinge domains. The kleisin subunit associates with the Smc2 and Smc4 ATPase head domains to create a ring-like structure and recruits two additional subunits (shown in gray, not studied here).

(B) Example image of Smc2-Smc4 dimers imaged by rotary shadowing EM.

(C) Example image of Smc2-Smc4 dimers imaged by dry AFM.

(D) Example images of different conformational classes of Smc2-Smc4 dimers from high-speed liquid AFM movies. The frequency of each conformational class (as fraction of 1,795 total frames from 18 movies) is indicated. V-shaped, SMCs are connected at the hinge but the heads are not engaged; O-shaped, the heads are engaged with each other; B-shaped (butterfly), both heads are engaged with the hinge; P-shaped, one of the heads is engaged with the hinge.

combination with supporting data from EM and dry AFM, to probe the structural arrangement and dynamics of condensin's Smc2-Smc4 dimers under physiological conditions. We show that the coiled coils are remarkably flexible, allowing the molecules to adopt various conformations that change over time. We furthermore find that, even in the absence of ATP or DNA, the heads of the Smc2 and Smc4 subunits dynamically engage with each other and with the Smc2-Smc4 hinge. Our findings show that condensin SMC dimers are able to adopt various conformations, which suggests that condensin complexes have the structural flexibility required to engage and link the chromatin fibers of eukaryotic genomes.

RESULTS

Smc2-Smc4 Dimers Display a Variety of Conformations

While this paper focuses on the results from liquid AFM, we first, as a point of reference and for comparison to reported data, used rotary shadowing EM to image Smc2-Smc4 dimers purified from budding yeast *Saccharomyces cerevisiae* (Figure 1B). Surprisingly, in only about one-third of the Smc2-Smc4 dimers, the coiled coils were closely juxtaposed over part or all of their lengths, resulting in the I- or Y-shaped conformations that were predicted from previous studies (Anderson et al., 2002; Soh et al., 2015; Yoshimura et al., 2002). The majority of molecules displayed instead clearly separated coiled coils, resulting in either V-shaped or O-shaped conformations (Figure S1A). In parallel to rotary shadowing EM, we also imaged the molecules by dry AFM (Figure 1C). Consistent with the EM data, the vast majority of molecules identified in the AFM images had separated coiled coils and appeared as V- or O-shaped dimers (Figure S1B). Notably, we never observed the coiled coils as juxtaposed stiff rods with dry AFM.

Since earlier EM and AFM studies investigated Smc2-Smc4 dimers of vertebrate and fission yeast condensin complexes, it is conceivable that the *S. cerevisiae* Smc2-Smc4 dimer represents an unusual exception to the previously reported rod-shaped architecture. We therefore purified and imaged another Smc2-Smc4 dimer, this time from the thermophilic yeast species *Chaetomium thermophilum* (Figure S1C). Similar to what we had observed for the *S. cerevisiae* Smc2-Smc4 dimer, we again found that the majority of the molecules were in either the V- or O-shaped conformations. We therefore conclude that the Smc2-Smc4 dimers of two yeast species, which diverged several hundred million years ago, can adopt a number of different conformations, with the majority in O or V shapes.

Since EM and dry AFM can only gather snapshots of protein conformations, we used high-speed AFM in liquid to create movies of *S. cerevisiae* Smc2-Smc4 dimers with a frame rate of ten frames per second. We classified the conformations of the dimers in over 1,700 frames taken with high-speed AFM (Figure 1D). The V-shaped conformation accounts for a quarter of the cases (Figure 1D, first row), while the most abundant configuration is the O-shaped conformation (Figure 1D, second row). Unexpectedly, liquid AFM imaging uncovered two additional conformations, which involve interactions between the head and hinge domains. In a conformation that we refer to as "butterfly" (B-shaped), both ATPase heads engage with the hinge and the intervening coils form two short loops that extrude from this head-hinge complex (Figure 1D, third row). In a conformation that we refer to as P-shaped conformation, only one of the heads engages with the hinge and the other head moves freely (Figure 1D, fourth row). We conclude that, in addition to the conformations also found with dry imaging techniques, high-speed AFM in liquid uncovered that Smc2-Smc4 dimers can adopt two additional conformations that had escaped prior notice.

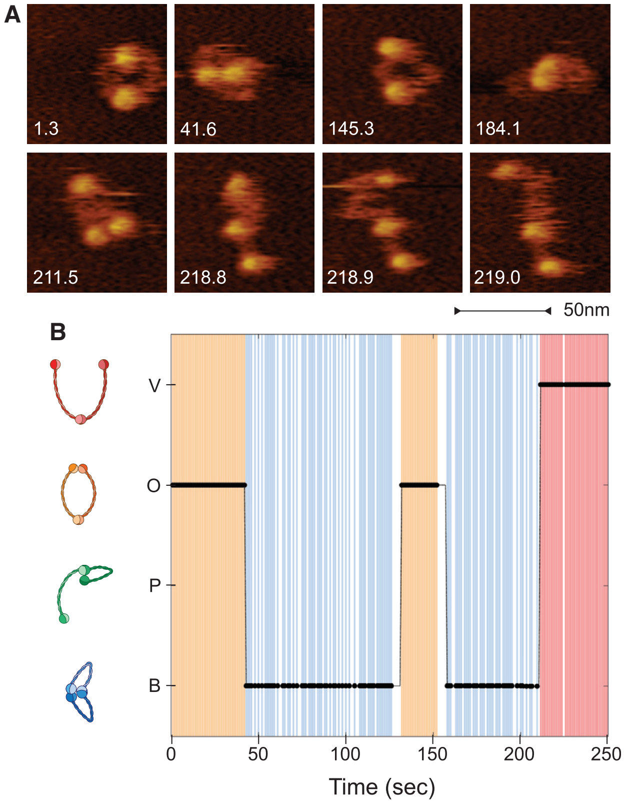

Figure 2. Smc2-Smc4 Dimers Change Conformations Dynamically
(A) Snapshots of an Smc2-Smc4 dimer followed over time with high-speed AFM at a rate of 10 frames per second in Movie S1. Snapshots are taken at various time points in the movie (shown in seconds).
(B) Annotation of conformational classes for each frame of Movie S1. O-shaped conformations are indicated in orange, B-shaped conformations in blue, V-shaped conformations in red. White gaps indicate that the conformation could not be confidently classified for a particular frame.

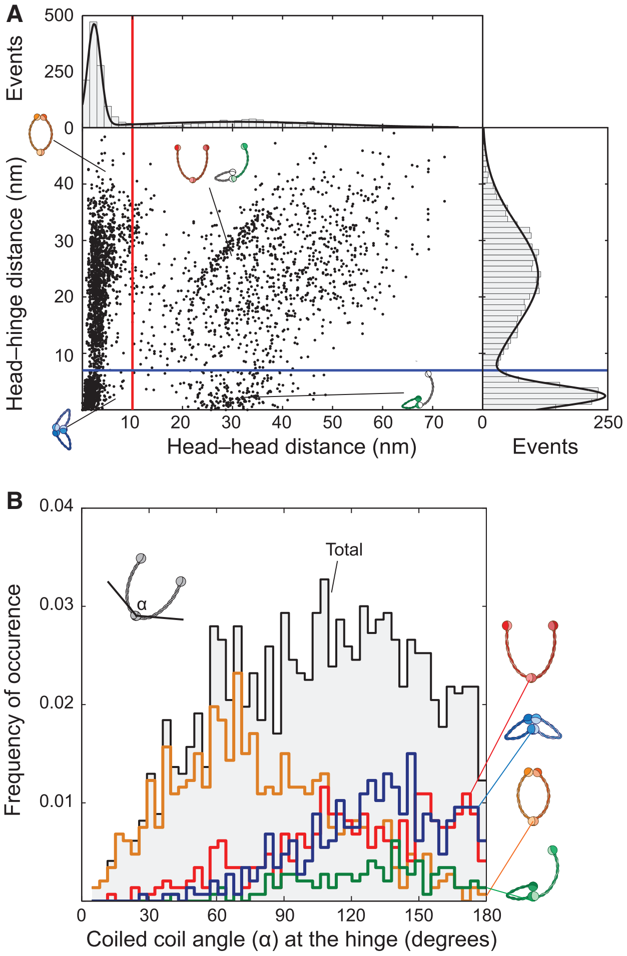

Figure 3. Structural Analysis of Smc2-Smc4 Conformations
(A) Scatterplot of head-head distances versus head-hinge distances for each subunit of an Smc2-Smc4 dimer imaged by high-speed liquid AFM (total 1,795 frames of 18 independent molecules). Each dot represents a measurement of one SMC subunit. Frequencies of data points for head-head distances or head-hinge distances are plotted as histograms at the top and right side of the plot, respectively. Data points left of the vertical red line and below the horizontal blue line: B-shaped conformations, points left of the red and above the blue line: O-shaped conformations, points right of the red and above the blue line: V-shaped conformations and the head-hinge disengaged arm of P-shaped conformations, points right of the red line and below the blue line: the head-hinge engaged arm of P-shaped conformations.
(B) Histogram plot of the angles between the Smc2-Smc4 coiled coils, measured at the hinge. The black histogram shows the frequency of occurrence for all conformations. Histograms for individual conformational classes are shown in red (V-shaped), orange (O-shaped), green (P-shaped), or blue (B-shaped).

Smc2-Smc4 Dimers Undergo Frequent Conformational Changes

Analysis of an individual Smc2-Smc4 dimer recorded in real time revealed that the dimer did not remain in one static configuration during the course of the experiment (Figure 2; Movie S1). At the start of the movie, the molecule was O-shaped (first frame in Figure 2A), and then the heads approached the hinge to form a "butterfly" structure (second frame in Figure 2A). The molecule switched between O- and B-shaped conformations multiple times before converting to a V-shaped "open" conformation toward the end of the movie (last four frames in Figure 2A). Remarkably, all Smc2-Smc4 dimers that we studied underwent conformational changes during the imaging time (Figure S2; Movies S1, S2, and S3).

Head-Head and Head-Hinge Engagements Are Dynamic

To more carefully analyze the dynamics by which the Smc2-Smc4 ATPase heads engage with each other and with the hinge, we determined the distances between the centers of the two heads and the distances between the centers of each head to the center of the hinge. The distance between the heads (Figure 3A, top histogram) showed a clear peak, which can be fit by a Gaussian profile at 2.5 ± 1.3 nm (error denotes SD). This peak corresponds to all conformations with associated head domains, i.e., all O- and B-shaped conformations, which group on the left side of the red line in the scatterplot (Figure 3A, main panel). The head-head distance distribution also contained a second population at much larger distances of 20–60 nm (right of the red line), which corresponds to V- and P-shaped

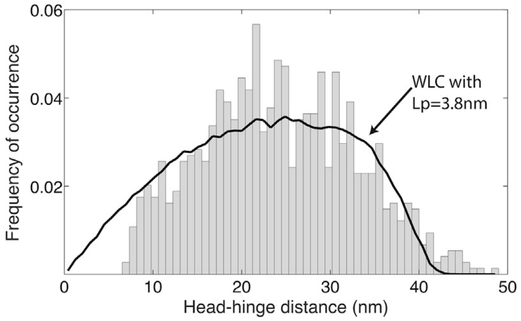

Figure 4. The Smc2-Smc4 Coiled Coils Can Be Characterized by a Worm-like Chain Model with a Persistence Length of ~4 nm

The measured end-to-end length histogram (head-hinge distance) of open configurations (gray bars) has a broad peak around 25 nm. This shape is well reproduced by the end-to-end distribution of 10^5 simulated worm-like-chain polymers with a persistence length of 3.8 nm (black line).

conformations. The distances between head and hinge domains (Figure 3A, right histogram) showed a large peak at 2.4 ± 1.9 nm. This peak corresponds to conformations in which at least one of the two heads engages with the hinge, i.e., all B- and P-shaped conformations in the group below the blue line in the scatterplot. The second broad peak at 23.8 ± 9.1 nm signals the large head-hinge distance in open V- and P-shaped conformations.

To quantify the degree of openness of the Smc2-Smc4 dimers, we measured the angle between the two coiled coils at the hinge. For all conformations combined (Figure 3B, black histogram), we find that the frequency of occurrence increases approximately linearly up to ~70 degrees and then levels off for higher angles. Low angles are strikingly absent, which reflects the fact that we never observed a conformation in which the coiled coils are clamped together into a rod. Furthermore, the angle distribution depends on the conformation of the dimer. In the O-shaped conformation, the frequency of occurrence has a broad asymmetric peak with a maximum near 70 degrees. In all other conformations, we observed almost exclusively large-angle conformations.

For comparison, we also measured the hinge angles of *S. cerevisiae* Smc2-Smc4 dimers in electron micrographs (Figure S3A). We again observed a wide distribution of angles between the two coils, with a peak at around 40 degrees and a lower occurrence of smaller angles. The quantitative difference between peak values measured by EM and liquid AFM implies that vacuum drying SMC dimers on mica surfaces, an unavoidable protocol for EM, may impact the coiled coil arrangement. To exclude the possibility that the attachment of the head domains to the surface artificially biases the coiled coils into an open conformation during preparation for EM, we also measured the hinge angles of "head-less" *C. thermophilum* Smc2-Smc4 dimers in electron micrographs (Figures S3B and S3D) and compared them to the angles measured for full-length *C. thermophilum* Smc2-Smc4 dimers (Figures S1C and S3C). In both cases, we again observed a wide distribution of angles with a peak around 40 degrees. These measurements confirm that the coiled coils emanate from the Smc2-Smc4 hinge domains in an open conformation, rather than in a juxtaposed closed conformation, independent of the presence of the ATPase head domains or species origin.

The SMC Coiled Coils Are Highly Flexible

A corollary of the finding that Smc2-Smc4 dimers can adopt a large number of conformations is that the coiled-coil structure of the SMC proteins must be very flexible and thereby allow the free movement of the head domains in relation to the hinge domain. In fact, the flexibility of the coils can be directly observed in the time-lapse recordings of the SMC dimers in liquid (Figure 2A; Movies S1, S2, and S3). Even when the molecule remains in the same conformational class, the coiled coils are highly mobile. For example, in the last three panels of the time lapse shown in Figure 2A, the Smc2–Smc4 dimer remains in the V-shaped conformation, but the coiled coils change their position between every frame (taken at 0.1-s intervals). The coils are even able to sharply bend into the B- and P-shaped conformations to enable head-hinge interactions (Figure 1D, third and forth rows), a motion that could not be achieved if the coiled coils were stiff.

We quantified this flexible behavior of the coiled coils by comparison to theoretical models developed for flexible polymers. The worm-like-chain (WLC) model is often used to describe the behavior of homogeneous semi-flexible polymers such as DNA or proteins (Bustamante et al., 1994; Kellermayer, 1997; van Noort et al., 2003). In the WLC model, the stiffness of a polymer is expressed as the persistence length L_P. It can be estimated from AFM images through the mean squared end-to-end distance of the coiled coil (i.e., head-hinge distance, Rivetti et al., 1996). We took only V-shaped conformations into account (to exclude the effect of the head-hinge and head-head interactions) and fitted WLC model predictions to the histogram of end-to-end lengths of the coiled coils (Figure S4A). Because there was no closed form available to describe this distribution analytically and approximations were only published for a limited set of persistence lengths (Hamprecht et al., 2004), we generated the distributions through Monte-Carlo simulations. These simulations reproduced the histogram of head-hinge distances for V-shaped conformations quite well. We found that our data are best described by a WLC model with an L_P of 3.8 ± 0.2 nm and a contour length of 46 ± 2 nm (Figure 4). As a visual control, we used the same simulation algorithm and parameters to generate example shapes of dimers. The simulations strikingly resemble our observations in high-speed AFM (Figure S4B), hereby confirming our method. We conclude that the coiled coils of Smc2-Smc4 dimers can be described as flexible polymers with a persistence length of only about 4 nm.

DISCUSSION

Using high-speed AFM under liquid conditions, we have examined the structure and dynamics of condensin's Smc2-Smc4 dimers in real time and under physiological conditions. Contrary to the suggestion from a crystal structure of the Smc2-Smc4 hinge domain, cross-linking experiments (Soh et al., 2015), and from images of Smc2-Smc4 dimers taken after drying them on a solid surface (Figure S1; Anderson et al., 2002; Yoshimura et al., 2002), we never observed rod-shaped molecules with their coiled coils juxtaposed under liquid conditions. We conclude

that the coiled coils are not stiff rods but are instead highly flexible.

Coiled coils serve a broad range of functions in many different proteins. It is hence useful to put our finding of the highly flexible nature of the Smc2-Smc4 coiled coils ($L_P \sim 4$ nm) into perspective. While a single alpha helix is very flexible ($L_P \sim 1$ nm; Papadopoulos et al., 2006), coiled coils are in general significantly more stiff. Theoretically, the persistence length of a coiled coil formed by two alpha helixes has been predicted to be as high as 200 nm (Yogurtcu et al., 2010). In reality, the global stiffness of a coiled-coil structure depends on its sequence and on local interruptions by non-coiled sequences. Measured values of L_P of coiled-coil proteins range from 25 nm for myosin II (Schwaiger et al., 2002) to up to 100 nm for tropomyosin (Li et al., 2010, 2012; Loong et al., 2012). All hitherto reported L_P values are larger than the value that we deduced for the Smc2-Smc4 dimers, emphasizing the remarkably flexible nature of these condensin subunits.

We find that Smc2-Smc4 dimers characteristically display an open structure. The discrepancy to previously reported structures can be due to several factors. First, to accurately assess the behavior of the coiled coils, it is important to take the full-length SMC proteins into account, since the engagement of the heads with each other and the hinge has an influence on the behavior of the coils. Our results indeed indicate that the heads have a certain attractive force toward each other and to the hinge. Measurements on truncated proteins that lack the heads or parts of the coiled coil could therefore yield skewed results. Second, sample preparation conditions for EM and dry AFM can result in experimental artifacts, such that certain conformations are missed. Measuring in liquid at near-physiological conditions is closer to the in vivo situation. Third, our liquid AFM data show that the configuration of the SMC dimers is dynamic over time. Consequently, results from bulk cross-linking experiments should be treated with caution, as transient interactions can be kinetically trapped with this method. Moreover, the open conformations would be missed and cross-linking interactions might occur between adjacent molecules.

We find that the ATPase heads of the Smc2-Smc4 dimers engage and disengage with each other in a dynamic manner. Excitingly, we find that the heads also interact dynamically with the hinge, resulting in a hitherto undiscovered structure. The dip in the head-hinge distance probability density at ~7 nm (Figure 3A, right panel) suggests that this interaction takes place despite a considerable entropic penalty. In the P-shaped form, only one head interacts with the hinge, which indicates that the two ATPase heads interact with the hinge independently of each other. It has previously been suggested that the head and hinge domains of cohesin's Smc1–Smc3 dimer might need to associate to enable an ATP hydrolysis-driven disengagement of the hinge for DNA entry into the cohesin ring, a feat that can only be achieved by the folding of the intervening coiled coils (Gruber et al., 2006; Nasmyth, 2011). If DNA enters condensin rings in an analogous manner to what has been proposed for cohesin, then the newly identified "butterfly" structure reveals a conformation that is important in condensin's DNA loading mechanism.

Here, we demonstrated that high-speed AFM in liquid is able to provide a quantitative analysis of the dynamics of SMC dimers under physiological conditions. We showed that even in the absence of ATP or DNA, Smc2-Smc4 dimers adopt highly dynamic and flexible conformations. The biophysical properties of the SMC coiled coils revealed by our study provide the fundamental basis for the mechanics of DNA entrapment by the SMC protein machinery and set the stage for further in-depth biochemical and structural studies of condensin and cohesin.

EXPERIMENTAL PROCEDURES

Purification of Smc2-Smc4 Dimers

S. cerevisiae Smc2 fused to a C-terminal His_6 epitope tag and Smc4 fused to a C-terminal StrepII tag were co-expressed from an episomal plasmid under the control of the galactose-inducible *GAL1* or *GAL10* promoters in protease-deficient budding yeast cells (strain C2598) and purified via Ni-NTA, StrepTactin and gel filtration steps as described (Piazza et al., 2014).

EM and Rotary Shadowing

Smc2–Smc4 protein preparations were dialyzed for 45 min against 200 mM NH_4HCO_3, 30% glycerol, and 2 mM DTT (pH 7.6). 3 μl of 0.1 mg/ml dialyzed Smc2-Smc4 dimers was sprayed onto freshly cleaved mica, immediately dried in vacuum, and rotary shadowed with Pt-C at an angle of 7°. Images were recorded in a Morgagni FEI microscope at 56,000× magnification.

Dry AFM

SMC dimers were diluted to a concentration of 7.1 μg/ml in 200 mM NaCl, 10 mM Tris-HCl (pH 7.0), 30 mM $MgCl_2$, 5 mM DTT, and 10% glycerol. Samples were incubated on mica for 10 s before rinsing with MilliQ water and drying with a nitrogen gun. Imaging was performed on a Bruker Multimode AFM, using BudgetSensors SHR150 ultrasharp probes.

High-Speed AFM in Liquid

Purified Smc2-Smc4 dimers at a concentration of 2.2 mg/ml were 20× diluted with imaging buffer (20 mM Tris-HCl [pH 7.0], 200 mM NaCl, 10 mM $MgCl_2$, 5 mM DTT, 10% glycerol) and immediately snap-frozen in aliquots and stored at –80°C. Prior to imaging, samples were thawed and diluted another 40 × with imaging buffer, and a droplet of protein solution was applied to freshly cleaved mica. After 10 s, the surface was rinsed with imaging buffer and placed—without drying—into the imaging bath of the AFM (HS-AFM 1.0, RIBM). Procedures for imaging were largely according to those described in published protocols (Uchihashi et al., 2012). Nanoworld USC-f1.2-k0.15 and USC-f1.5-k-0.6 cantilevers were used. AFM movies of selected areas with single molecules, typically 70 × 80 nm in size, were acquired at frame rates of 2–10 Hz. The tip forces are controlled through a system that stabilizes the oscillation via a feedback mechanism on the second harmonic amplitude (Kodera et al., 2006; Schiener et al., 2004).

Image Analysis

A user-guided semi-automatic image analysis was performed. Because of the large data volume, only every fifth frame of the AFM movies was analyzed. This resulted in a total of more than 1,700 data points.

Stiffness Analysis and Monte Carlo Simulations

We simulated two-dimensional worm-like chains by dividing each chain into N segments of length $L_s = 0.2$ nm, and assigning angles α between the segments that are drawn from a normal distribution with a variance L_p/L_s. This definition of the persistence length L_p follows the analysis of Rivetti et al. (1996). From the angles between segments, x and y coordinates are calculated for the entire chain. For each value of the persistence length and contour length, 10^6 chains are simulated (see Figure S4B for examples), and histograms are calculated of the end-to-end distances. Using the sum of the least-squares between the Monte Carlo results and the experimental data as a goodness-of-fit estimator, the values of L_p and L_c are iterated to obtain the best fits and confidence intervals. The errors of the fits are one SD confidence intervals, obtained through the graphical Monte Carlo method (Cowan, 1998). We note that this procedure significantly extends beyond the traditional approach first described by Rivetti et al., which yields an estimate for the persistence length using only the mean

square end-to-end distance. In our case, that approach yields a value of 4.6 nm. The difference can be ascribed to the fact that data for end-to-end distances near zero are missing from the distribution due to the head-hinge interactions. Finally, it should be noted that in our experimental data, the centers of the heads and hinges are taken as markers for the ends of the coiled coils; i.e., we do not take the finite size of the hinge and head domains into account, nor the (unknown) position of the attachment points.

SUPPLEMENTAL INFORMATION

Supplemental Information includes Supplemental Experimental Procedures, four figures, one table, and three movies and can be found with this article online at http://dx.doi.org/10.1016/j.celrep.2016.01.063.

AUTHOR CONTRIBUTIONS

Conceptualization, J.M.E., A.J.K., C.H.H., and C.D.; Formal Analysis, J.M.E., A.J.K., and C.H.H.; Software, J.M.E. and A.J.K.; Investigation, A.J.K., E.M.D., L.d.W., M.K., M.H., and C.H.H.; Writing, J.M.E., A.J.K., C.H.H., and C.D.; Supervision, C.H.H. and C.D.

ACKNOWLEDGMENTS

We thank Helmut Schiessel for helpful discussions. We are grateful to the EMBL Electron Microscopy Core Facility for assistance. This work was supported by the ERC Advanced Grant SynDiv (no. 669598 to C.D.), by the Netherlands Organization for Scientific Research (NWO/OCW) as part of the Frontiers of Nanoscience program, by EMBL, and by the German Research Foundation (Grant HA5853/2-1 to C.H.H.).

Received: October 14, 2015
Revised: December 21, 2015
Accepted: January 20, 2016
Published: February 18, 2016

REFERENCES

Anderson, D.E., Losada, A., Erickson, H.P., and Hirano, T. (2002). Condensin and cohesin display different arm conformations with characteristic hinge angles. J. Cell Biol. *156*, 419–424.

Ando, T., Kodera, N., Takai, E., Maruyama, D., Saito, K., and Toda, A. (2001). A high-speed atomic force microscope for studying biological macromolecules. Proc. Natl. Acad. Sci. USA *98*, 12468–12472.

Aragon, L., Martinez-Perez, E., and Merkenschlager, M. (2013). Condensin, cohesin and the control of chromatin states. Curr. Opin. Genet. Dev. *23*, 204–211.

Barysz, H., Kim, J.H., Chen, Z.A., Hudson, D.F., Rappsilber, J., Gerloff, D.L., and Earnshaw, W.C. (2015). Three-dimensional topology of the SMC2/SMC4 subcomplex from chicken condensin I revealed by cross-linking and molecular modelling. Open Biol. *5*, 150005.

Bustamante, C., Marko, J.F., Siggia, E.D., and Smith, S. (1994). Entropic elasticity of lambda-phage DNA. Science *265*, 1599–1600.

Cowan, G. (1998). Statistical Data Analysis (Oxford University Press).

Cuylen, S., Metz, J., and Haering, C.H. (2011). Condensin structures chromosomal DNA through topological links. Nat. Struct. Mol. Biol. *18*, 894–901.

Gruber, S., Arumugam, P., Katou, Y., Kuglitsch, D., Helmhart, W., Shirahige, K., and Nasmyth, K. (2006). Evidence that loading of cohesin onto chromosomes involves opening of its SMC hinge. Cell *127*, 523–537.

Haering, C.H., Löwe, J., Hochwagen, A., and Nasmyth, K. (2002). Molecular architecture of SMC proteins and the yeast cohesin complex. Mol. Cell *9*, 773–788.

Haering, C.H., Farcas, A.-M., Arumugam, P., Metson, J., and Nasmyth, K. (2008). The cohesin ring concatenates sister DNA molecules. Nature *454*, 297–301.

Hamprecht, B., Janke, W., and Kleinert, H. (2004). End-to-end distribution function of two-dimensional stiff polymers for all persistence lengths. Phys. Lett. A *330*, 254–259.

Hirano, T. (2006). At the heart of the chromosome: SMC proteins in action. Nat. Rev. Mol. Cell Biol. *7*, 311–322.

Huis in 't Veld, P.J., Herzog, F., Ladurner, R., Davidson, I.F., Piric, S., Kreidl, E., Bhaskara, V., Aebersold, R., and Peters, J.-M. (2014). Characterization of a DNA exit gate in the human cohesin ring. Science *346*, 968–972.

Katan, A.J., and Dekker, C. (2011). High-speed AFM reveals the dynamics of single biomolecules at the nanometer scale. Cell *147*, 979–982.

Kellermayer, M.S. (1997). Folding-unfolding transitions in single titin molecules characterized with laser tweezers. Science *276*, 1112–1116.

Kodera, N., Sakashita, M., and Ando, T. (2006). Dynamic proportional-integral-differential controller for high-speed atomic force microscopy. Rev. Sci. Instrum. *77*, 083704.

Li, X.E., Holmes, K.C., Lehman, W., Jung, H., and Fischer, S. (2010). The shape and flexibility of tropomyosin coiled coils: implications for actin filament assembly and regulation. J. Mol. Biol. *395*, 327–339.

Li, X.E., Suphamungmee, W., Janco, M., Geeves, M.A., Marston, S.B., Fischer, S., and Lehman, W. (2012). The flexibility of two tropomyosin mutants, D175N and E180G, that cause hypertrophic cardiomyopathy. Biochem. Biophys. Res. Commun. *424*, 493–496.

Loong, C.K.P., Zhou, H.-X., and Chase, P.B. (2012). Persistence length of human cardiac α-tropomyosin measured by single molecule direct probe microscopy. PLoS ONE *7*, e39676.

Nasmyth, K. (2011). Cohesin: a catenase with separate entry and exit gates? Nat. Cell Biol. *13*, 1170–1177.

Papadopoulos, P., Floudas, G., Schnell, I., Lieberwirth, I., Nguyen, T.Q., and Klok, H.-A. (2006). Thermodynamic confinement and alpha-helix persistence length in poly(gamma-benzyl-L-glutamate)-b-poly(dimethyl siloxane)-b-poly (gamma-benzyl-L-glutamate) triblock copolymers. Biomacromolecules *7*, 618–626.

Piazza, I., Rutkowska, A., Ori, A., Walczak, M., Metz, J., Pelechano, V., Beck, M., and Haering, C.H. (2014). Association of condensin with chromosomes depends on DNA binding by its HEAT-repeat subunits. Nat. Struct. Mol. Biol. *21*, 560–568.

Rivetti, C., Guthold, M., and Bustamante, C. (1996). Scanning force microscopy of DNA deposited onto mica: equilibration versus kinetic trapping studied by statistical polymer chain analysis. J. Mol. Biol. *264*, 919–932.

Schiener, J., Witt, S., Stark, M., and Guckenberger, R. (2004). Stabilized atomic force microscopy imaging in liquids using second harmonic of cantilever motion for setpoint control. Rev. Sci. Instrum. *75*, 2564–2568.

Schwaiger, I., Sattler, C., Hostetter, D.R., and Rief, M. (2002). The myosin coiled-coil is a truly elastic protein structure. Nat. Mater. *1*, 232–235.

Soh, Y.-M., Bürmann, F., Shin, H.-C., Oda, T., Jin, K.S., Toseland, C.P., Kim, C., Lee, H., Kim, S.J., Kong, M.-S., et al. (2015). Molecular basis for SMC rod formation and its dissolution upon DNA binding. Mol. Cell *57*, 290–303.

Uchihashi, T., Kodera, N., and Ando, T. (2012). Guide to video recording of structure dynamics and dynamic processes of proteins by high-speed atomic force microscopy. Nat. Protoc. *7*, 1193–1206.

van Noort, J., van Der Heijden, T., de Jager, M., Wyman, C., Kanaar, R., and Dekker, C. (2003). The coiled-coil of the human Rad50 DNA repair protein contains specific segments of increased flexibility. Proc. Natl. Acad. Sci. USA *100*, 7581–7586.

Yogurtcu, O.N., Wolgemuth, C.W., and Sun, S.X. (2010). Mechanical response and conformational amplification in α-helical coiled coils. Biophys. J. *99*, 3895–3904.

Yoshimura, S.H., Hizume, K., Murakami, A., Sutani, T., Takeyasu, K., and Yanagida, M. (2002). Condensin architecture and interaction with DNA: regulatory non-SMC subunits bind to the head of SMC heterodimer. Curr. Biol. *12*, 508–513.

Cell Reports, Volume *14*

Supplemental Information

Condensin Smc2-Smc4 Dimers Are Flexible and Dynamic

Jorine M. Eeftens, Allard J. Katan, Marc Kschonsak, Markus Hassler, Liza de Wilde, Essam M. Dief, Christian H. Haering, and Cees Dekker

Figure S1

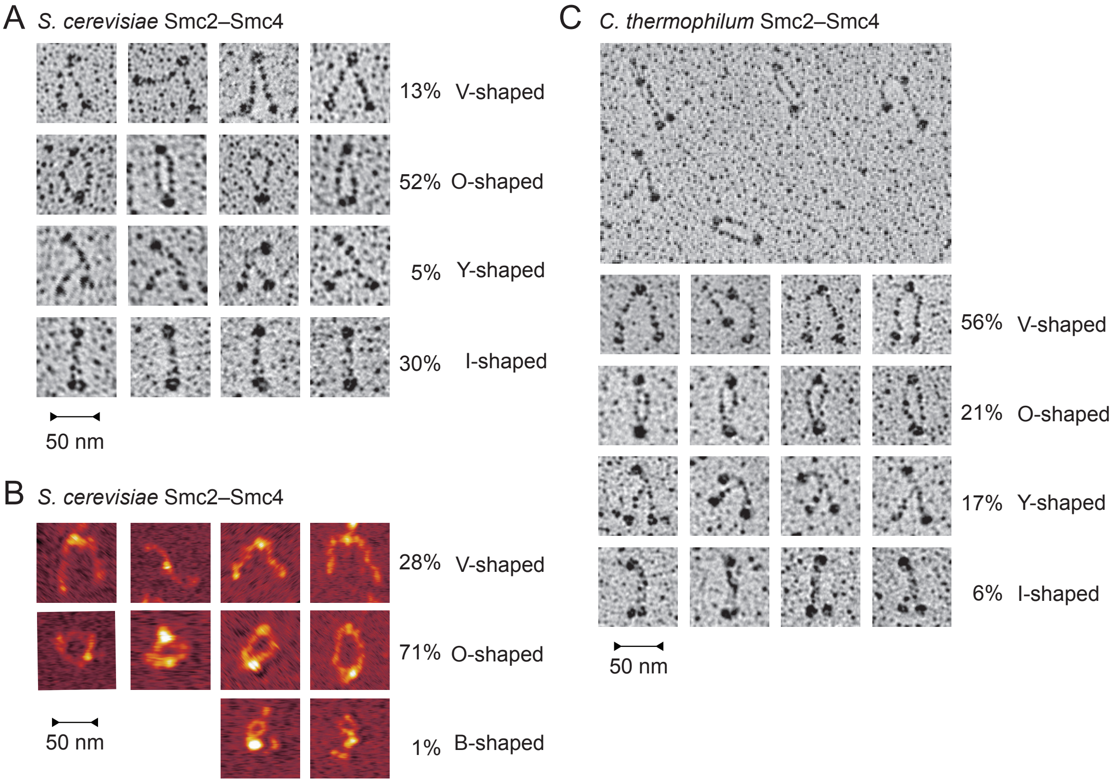

Figure S1: Smc2–Smc4 dimer conformations in EM and dry AFM, related to Figure 1

(A) Examples of different conformation classes of *S. cerevisiae* Smc2–Smc4 dimers observed by rotary shadowing EM. Frequencies of occurrence are listed on the right as a fraction of 500 molecules classified. Molecules were categorized as O-shaped if a space of at least the width of a coiled coil was visible between the SMC coiled coils. **(B)** Examples of different conformations of *S. cerevisiae* Smc2–Smc4 dimers observed by dry AFM. Frequencies of occurrence are listed on the right as a fraction of 400 molecules classified. **(C)** Examples of different conformations of *Ch. thermophilum* Smc2–Smc4 dimers observed by rotary shadowing EM. Frequencies of occurrence are listed on the right as a fraction of 400 molecules classified.

Figure S2

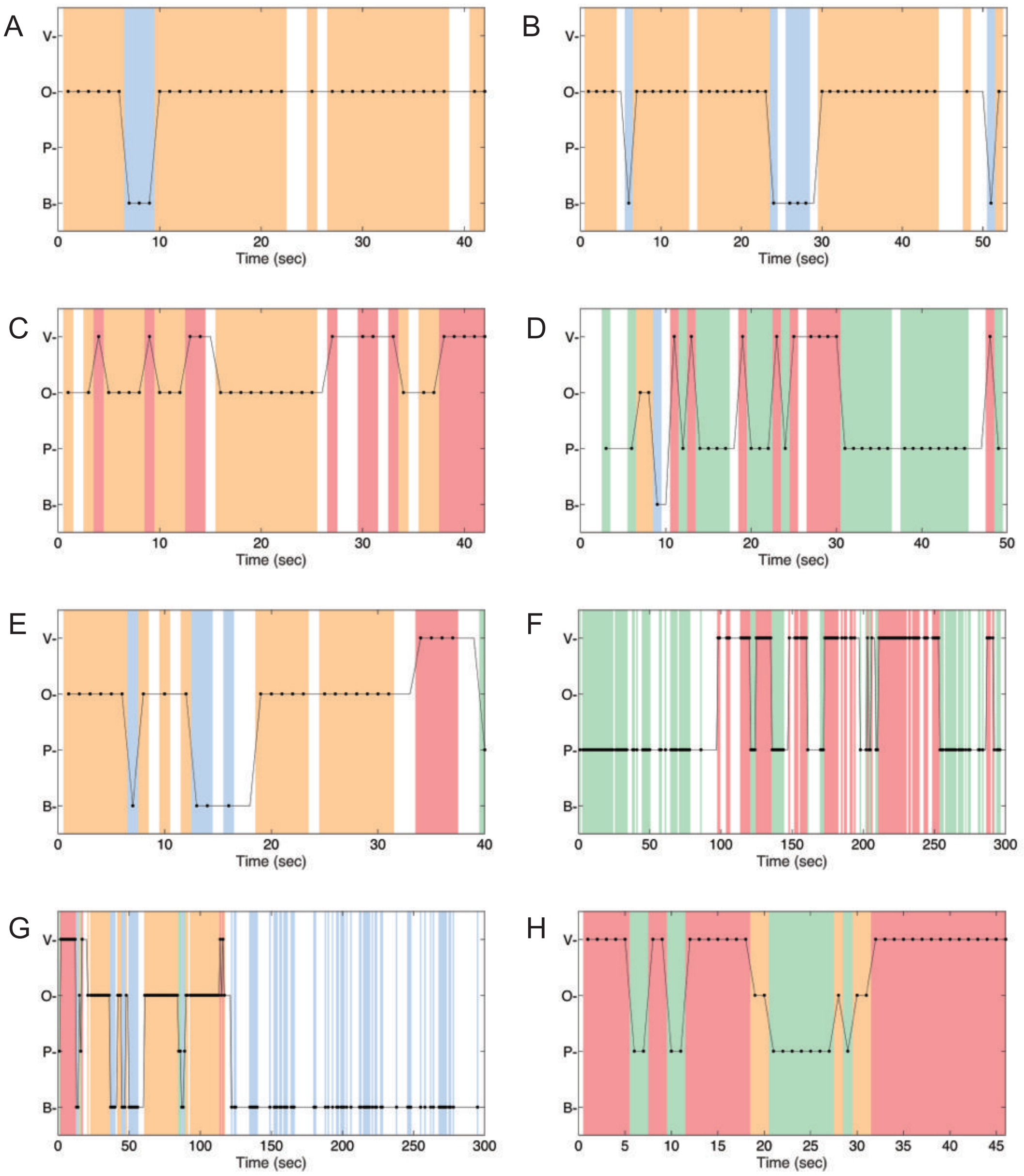

Figure S2: Conformational changes of different Smc2–Smc4 dimers followed by high-speed AFM, related to Figure 2

Conformations of individual frames of eight movies of different Smc2–Smc4 molecules were classified. V-shaped in red, O-shaped in orange, P-shaped in green, B-shaped in blue. White gaps indicate that the conformation could not be confidently classified for a particular frame.

Figure S3

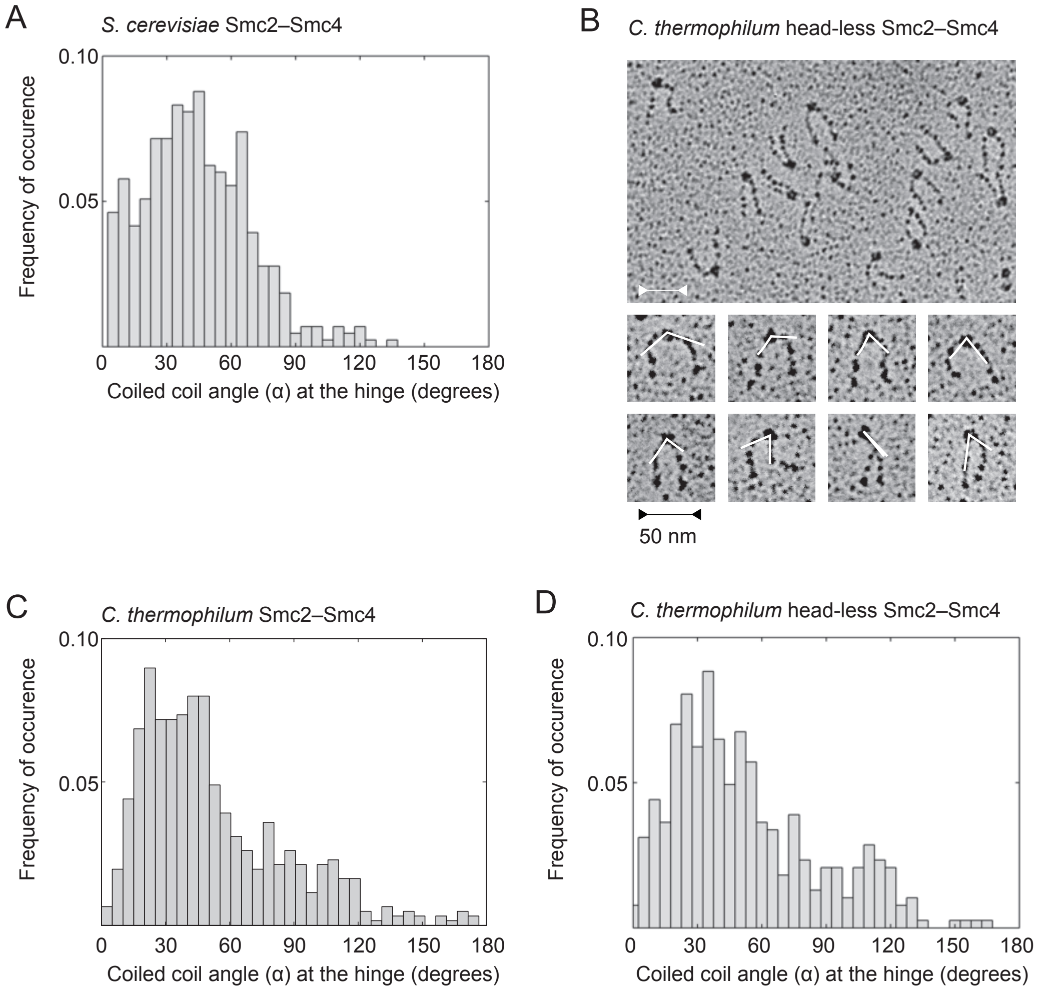

Figure S3: Coiled coil angle measured in electron micrographs, related to Figure 3

(A) Histogram frequency plot of coiled-coil angles measured at the hinge of full-length *S. cerevisiae* Smc2–Smc4 dimers (displayed as fraction of a total 430 individual molecules). **(B)** Example image of ‘head-less *Ch. thermophilum* Smc2-Smc4 dimers imaged by rotary shadowing EM. **(C)** Histogram frequency plot of the coiled coil angles at the hinge of head-less *Ch. Thermophilum* Smc2–Smc4 dimers (displayed as fraction of a total 385 individual molecules). **(D)** Histogram frequency plot of the coiled coil angles at the hinge *Ch. Thermophilum* Smc2–Smc4 dimers (displayed as fraction of a total 613 individual molecules).

Figure S4

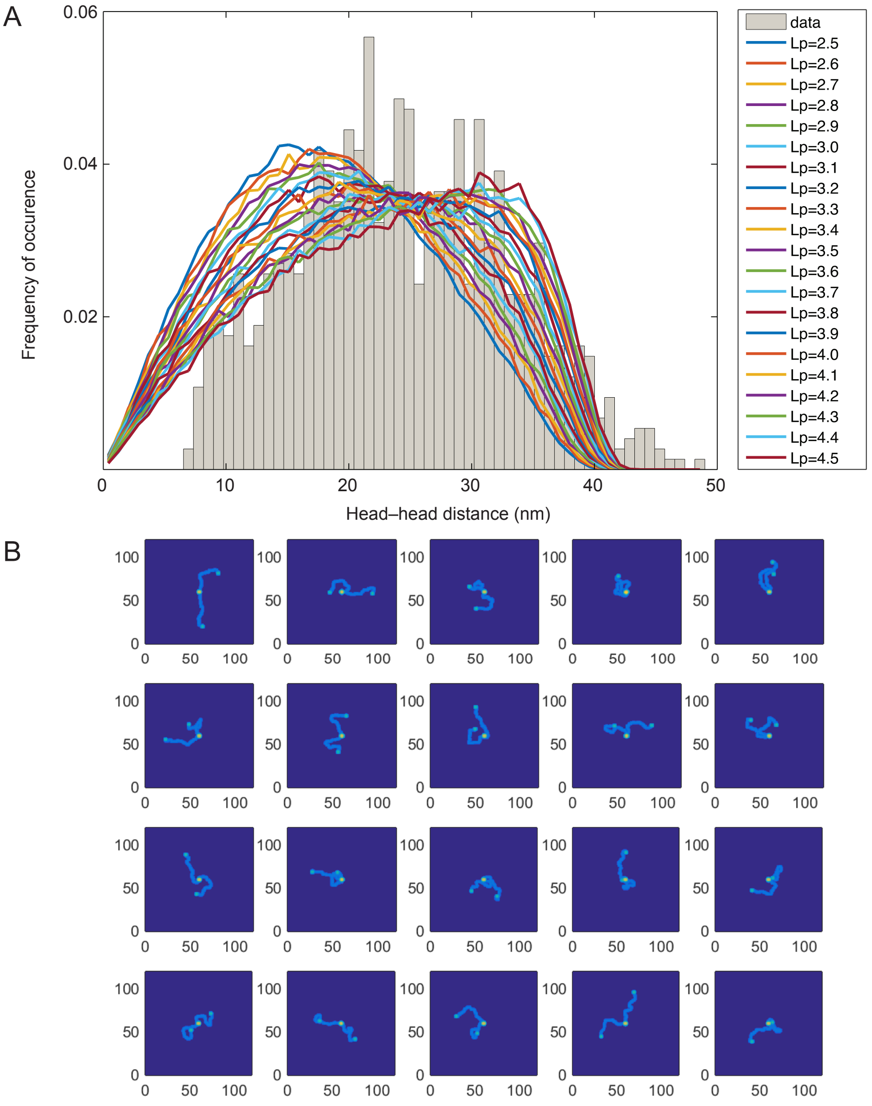

Figure S4: A worm-like chain model to describe the behavior of Smc2–Smc4 dimers, related to Figure 4

(A) We simulated worm-like chains with varying persistence length and a set contour length of 46 nm. A persistence length of 3.8 nm fits our data measured for *S. cerevisiae* Smc2–Smc4 dimers in the V-shaped (grey histogram) best, as judged from minimizing for the least squared residues. **(B)** We used the found persistence length of 3.8 nm to simulate the behavior of the coiled-coils. The results of these simulations resemble our AFM images.

Table S1:

Persistence lengths of coiled coils and other biological polymers, ranged from stiff to flexible, related to Figure 4.

**Polymer**	**Persistence length**	**Reference**
Coiled coil (theorerical)	200nm	Yogurtcu et al., 2010
Tropomyosin coiled coil	40-100nm	Li et al., 2010, 2012; Loong et al., 2012
Vimentin coiled coil	63nm	Ackbarow and Buehler, 2007
Double stranded RNA	62nm	Abels et al., 2005
Double stranded DNA	50nm	Bustamante et al., 1994
Rad50 coiled coil	35nm	de Jager et al., 2001
Myosin II coiled coil	25nm	Schwaiger et al., 2002
Smc2-Smc4 coiled coil	*4nm*	*this manuscript*
Protein alpha helix	1nm	Papadopoulos et al., 2006
Single strand DNA	0.8nm	Smith et al., 1996
Unstructured amino acid chain	0.4nm	Carrion-Vazquez et al., 1999

Supplemental Movies

Movie S1: Movie of an Smc2-Smc4 dimer taken with high-speed AFM, related to Figure 2.

This movie corresponds to the time trace in Figure 2B.

Movie S2: Movie of an Smc2–Smc4 dimer taken with high-speed AFM, related to Figure 2.

This movie corresponds to the time trace in Figure S2F.

Movie S3: Movie of an Smc2-Smc4 dimer taken with high-speed AFM, related to Figure 2.

This movie corresponds to the time trace in Figure S2G.

Supplemental experimental procedures

Force control in AFM imaging and the possible influence of the scanning motion on SMC dynamics

We operate the AFM in intermittent contact ('tapping') mode. The tapping amplitude A during imaging is approximately 4 nm, and the 'amplitude ratio' (imaging amplitude divided by free amplitude) is typically 85-95%. The imaging feedback keeps the amplitude A constant, but because the drive efficiency of the tapping piezo is subject to temporal fluctuations, the amplitude ratio can fluctuate substantially outside the user's control. To have more control over the applied forces, A_2, the second harmonic of the cantilever's sinusoidal motion is monitored using a Signal Recovery model 7280 lock-in amplifier and kept constant by a homebuilt feedback unit, that modulates the drive amplitude. In the range of imaging parameters that we use, A_2 is monotonic in the strength of the tip-sample interaction, and therefore it is a good proxy for the imaging force. However, the exact relation between A_2 and force depends on several unknown parameters and is therefore not quantifiable.

The bandwidth of the AFM imaging system depends on the force that is applied to the sample. At the lowest imaging forces, the image quality is poor and only the location, but not the conformation of the dimers can be seen. Evaluation of these images indicates that the mobility of the dimers is not reduced under these imaging conditions compared to the higher forces used to obtain quantitative results, suggesting that the tip influence on these results is minimal. To obtain images that are sharp enough to distinguish all four configurations from each other while maintaining an image speed of 2 frames/s, we find that an A_2 value of 20 pm or more is required. Values of 50-70 pm are sufficient to obtain high-quality image*s* up to 10 frames/s, and hence we did not go beyond this. Notably, within the range of forces that yields movies of sufficient quality, we find no effect of the imaging on the dynamics of the SMC dimers. In addition, we see no correlation between the head positions and the fast scanning direction. We therefore conclude that there is no indication that the AFM scanning induces changes to the molecular configuration of the SMC dimers or the dynamic behavior of the heads through energy input from the tip.

Purification of full-length Chaetomium thermophilum Smc2-Smc4 dimers

C. thermophilum Smc2 fused to a C-terminal StrepII tag and Smc4 (residues 1-1,542) fused to a C-terminal His_8 tag were co-expressed in Sf21 cells cultured in Sf-900 III SFM serum-free medium (Invitrogen) from a single baculovirus. About 0.5-0.8×10^8 Sf21 cells were lysed by sonication in lysis buffer (50mM TRIS-HCl pH 7.5, 300mM NaCl, 20mM imidazole, 5mM β-mercaptoethanol) supplemented with 1× Complete EDTA-free protease inhibitor cocktail (Roche) at 4˚C. The lysate was cleared by centrifugation at 45,000×g for 50min at 4˚C and incubated with NiNTA Sepharose (GE Healthcare) for 2h at 4˚C. The resin was washed with ~100 column volumes (CV) lysis buffer before elution with 4 CV elution buffer (50mM TRIS-

HCl pH 7.5, 300mM NaCl, 320mM imidazole, 5mM β-mercaptoethanol). Eluted protein fractions were combined and dialyzed over night at 4˚C in dialysis buffer (25mM TRIS-HCl pH 7.5, 300mM NaCl, 1mM DTT), diluted with 10 volumes of low-salt buffer (25mM TRIS-HCl pH 7.5, 100mM NaCl, 1mM DTT), and loaded onto a 6ml-RESOURCE Q anion-exchange column (GE Healthcare). The resin was washed with 5 CV low-salt buffer and eluted in a 60ml linear gradient to high-salt buffer (25mM TRIS-HCl pH 7.5, 1 M NaCl, 1mM DTT). Peak fractions were collected and concentrated by ultrafiltration with a 100,000 MWCO cut-off Vivaspin concentrator (Sartorius).

Purification of head-less Chetomium Thermophilum Smc2-Smc4 dimers

N-terminally His_6-tagged Smc2 (residues 224-982) and Smc4 (residues 509-1,258) from *C. thermophilum* were co-expressed in *E. coli* strain BL21(DE3)Rosetta2 pLysS (Novagen) grown in 2×TY medium and induced with 200μM IPTG at 18°C for 16h. Cell pellets were resuspended in lysis buffer (50mM TRIS-HCl pH 7.5, 500mM NaCl, 25 mM imidazole, 5mM β-mercaptoethanol) supplemented with 1× Complete EDTA-free protease inhibitors and lysed using a sonicator (Branson). Cell debris were pelleted for 1h at 40,000×g and the supernatant was incubated with NiNTA Sepharose (GE Healthcare) pre-equilibrated in lysis buffer. The resin was extensively washed with lysis buffer and eluted with 250mM imidazole. Eluted protein was dialyzed to 50mM TRIS-HCl pH 7.5, 100mM NaCl, 1mm DTT and then loaded onto a RESOURCE Q anion-exchange column equilibrated in the same buffer. Proteins were eluted with a linear gradient to 1M NaCl. The single peak fraction was concentrated and passed over a Superdex S200 26/60 gel filtration column (GE Healthcare) in 25mM TRIS pH 7.5, 200mM NaCl, 1mM DTT, where the proteins eluted in a single peak. Peak fractions were concentrated using a Vivaspin concentrator, flash frozen in liquid nitrogen, and stored at -80°C.

Supplemental references

Abels, J.A., Moreno-Herrero, F., van der Heijden, T., Dekker, C., and Dekker, N.H. (2005). Single-molecule measurements of the persistence length of double-stranded RNA. Biophys. J. *88*, 2737–2744.

Ackbarow, T., and Buehler, M.J. (2007). Superelasticity, energy dissipation and strain hardening of vimentin coiled-coil intermediate filaments: atomistic and continuum studies. J. Mater. Sci. *42*, 8771–8787.

Brown André, E.X., Litvinov, R.I., Discher, D.E., and Weisel, J.W. (2007). Forced Unfolding of Coiled-Coils in Fibrinogen by Single-Molecule AFM. Biophys. J. *92*, L39–L41.

Bustamante, C., Marko, J.F., Siggia, E.D., and Smith, S. (1994). Entropic elasticity of lambda-phage DNA. Science *265*, 1599–1600.

Carrion-Vazquez, M., Oberhauser, A.F., Fowler, S.B., Marszalek, P.E., Broedel, S.E., Clarke, J., and Fernandez, J.M. (1999). Mechanical and chemical unfolding of a single protein: A comparison. Proc. Natl. Acad. Sci. *96*, 3694–3699.

de Jager, M., van Noort, J., van Gent, D.C., Dekker, C., Kanaar, R., and Wyman, C. (2001). Human Rad50/Mre11 Is a Flexible Complex that Can Tether DNA Ends. Mol. Cell *8*, 1129–1135.

Li, X.E., Holmes, K.C., Lehman, W., Jung, H., and Fischer, S. (2010). The shape and flexibility of tropomyosin coiled coils: implications for actin filament assembly and regulation. J. Mol. Biol. *395*, 327–339.

Li, X.E., Suphamungmee, W., Janco, M., Geeves, M.A., Marston, S.B., Fischer, S., and Lehman, W. (2012). The flexibility of two tropomyosin mutants, D175N and E180G, that cause hypertrophic cardiomyopathy. Biochem. Biophys. Res. Commun. *424*, 493–496.

Loong, C.K.P., Zhou, H.-X., and Chase, P.B. (2012). Persistence length of human cardiac α-tropomyosin measured by single molecule direct probe microscopy. PLoS One *7*, e39676.

Papadopoulos, P., Floudas, G., Schnell, I., Lieberwirth, I., Nguyen, T.Q., and Klok, H.-A. (2006). Thermodynamic confinement and alpha-helix persistence length in poly(gamma-benzyl-L-glutamate)-b-poly(dimethyl siloxane)-b-poly(gamma-benzyl-L-glutamate) triblock copolymers. Biomacromolecules *7*, 618–626.

Rief, M., Pascual, J., Saraste, M., and Gaub, H.E. (1999). Single molecule force spectroscopy of spectrin repeats: low unfolding forces in helix bundles. J. Mol. Biol. *286*, 553–561.

Smith, S.B., Cui, Y., and Bustamante, C. (1996). Overstretching B-DNA: The Elastic Response of Individual Double-Stranded and Single-Stranded DNA Molecules. Science (80-.). *271*, 795–799.
